# Arginine-dependent immune responses

**DOI:** 10.1007/s00018-021-03828-4

**Published:** 2021-05-26

**Authors:** Adrià-Arnau Martí i Líndez, Walter Reith

**Affiliations:** Department of Pathology and Immunology, Faculty of Medicine, University of Geneva, Geneva, Switzerland

**Keywords:** Arginine metabolism, Nitric oxide synthase, Arginase, Immunity, Immunometabolism, Arginase 2, Arginase 1, NOS

## Abstract

A growing body of evidence indicates that, over the course of evolution of the immune system, arginine has been selected as a node for the regulation of immune responses. An appropriate supply of arginine has long been associated with the improvement of immune responses. In addition to being a building block for protein synthesis, arginine serves as a substrate for distinct metabolic pathways that profoundly affect immune cell biology; especially macrophage, dendritic cell and T cell immunobiology. Arginine availability, synthesis, and catabolism are highly interrelated aspects of immune responses and their fine-tuning can dictate divergent pro-inflammatory or anti-inflammatory immune outcomes. Here, we review the organismal pathways of arginine metabolism in humans and rodents, as essential modulators of the availability of this semi-essential amino acid for immune cells. We subsequently review well-established and novel findings on the functional impact of arginine biosynthetic and catabolic pathways on the main immune cell lineages. Finally, as arginine has emerged as a molecule impacting on a plethora of immune functions, we integrate key notions on how the disruption or perversion of arginine metabolism is implicated in pathologies ranging from infectious diseases to autoimmunity and cancer.

## Introduction

The restriction of amino acid availability is an evolutionarily conserved strategy for controlling cellular functions of both the host and pathogens [1]. One such amino acid is arginine, a proteinogenic *α*-amino acid that is encrypted in mRNA molecules for protein synthesis. As a free amino acid, arginine serves as the substrate for different nitrogen-containing compounds. In ureotelic animals, arginine is a key substrate for ammonia detoxification via the urea cycle. Free arginine also serves as a substrate for other biologically active compounds. Being a core metabolic module, arginine metabolism directly and indirectly participates in a plethora of biological phenomena, such as vasodilation, calcium release, regeneration of adenosine triphosphate, neurotransmission, cell proliferation, and, most notably, immunity [2, 3]. Beyond constituting a mere nutrient, arginine metabolism has recently emerged as a critical pathway for controlling immune cell function, including metabolically demanding activated T cells [4, 5]. Certain pathogens and malignant cells exploit arginine’s relevance for the immune system and co-opt arginine metabolism to impede host immune responses [6].

## Arginine: a multifaceted amino acid

Arginine is defined as a semi-essential amino acid, because under certain conditions, such as growth during infancy, pregnancy, severe immune challenge, or burn injuries [7, 8]; humans require a supplemental intake of dietary arginine. Human adults are able to synthesize arginine from glutamine, glutamate, and proline [9, 10], and dietary amino acids represent the major influx of circulating arginine. However, endogenous arginine synthesis does not suffice to compensate for dietary arginine insufficiency [11, 12]. The dependence on dietary arginine provision during periods of increased demand for this amino acid consequently accounts for the conditional essentiality of arginine [13].

Arginine was first isolated in 1886 [14] and identified as a proteinogenic amino acid soon afterwards [15]. In 1932, Krebs and Henseleit first postulated the existence of the urea cycle [16], pioneering the identification of a prominent role of l-arginine (hereafter arginine) in human physiology: the detoxification of neurotoxic ammonia [17]. Besides protein anabolism and ammonia detoxification, arginine regulates a plethora of biological processes, such as vasodilation, calcium signaling, regeneration of adenosine triphosphate, neurotransmission, cell proliferation, and immunity [2, 3].

Arginine serves as a precursor for numerous biologically active compounds: nitric oxide (NO), ornithine, proline and polyamines, creatine and hence phosphocreatine, and agmatine. Arginine also regulates its synthesis via allosteric activation of the synthesis of *N*-acetylglutamate [18], a cofactor subsequently required for glutamine metabolization into arginine. Moreover, infused arginine—but not enteral arginine—acts as a secretagogue, stimulating the secretion of anabolic hormones, such as insulin, glucagon, prolactin, somatostatin, pancreatic polypeptide from the pancreas, and adrenal catecholamines [19–21]. In support of its endocrine effects, arginine transport into pancreatic beta-cells leads to membrane depolarization and electrochemical stimulation of beta-cells [19].

The p*K*_a_ value of 13.8 ± 0.1 of arginine’s guanidinium group [22] implies that the arginine side chain remains protonated throughout all physiologic conditions, therefore, operating as a positively charged, basic amino acid. Moreover, it has been hypothesized that the positively charged guanidium group interferes with the incorporation of arginine into the hydrophobic interior of proteins, exerting an evolutionary selective pressure against the incorporation of arginine into bigger and more complex proteins [23]. Paradoxically, although the genetic code comprises six codons for arginine incorporation into proteins, the average content of arginine in animal proteins is markedly lower than what would be expected from the theoretical frequency of codons associated to it [24]. This negative bias against arginine incorporation into proteins could conceal a repurposing of arginine beyond structural functions, implying the evolution of free-arginine usage towards regulatory functions. In this respect, a growing body of evidence indicates that over the course of immune system evolution, arginine has been selected as a metabolic node for the regulation of immune responses [4].

## Potential of arginine as an immune enhancer

Early studies established that arginine availability has critical roles in the immune system. Detrimental effects of arginine starvation on human T lymphocytes were first described in 1968 by the establishment of a causal relationship between arginine depletion and impaired in vitro activation of lymphocytes stimulated with phytohemagglutinin [25]. Subsequent in vitro studies demonstrated that human Burkitt lymphoma B cells also require adequate arginine concentrations for their proliferation and maturation [26]. Animal experimentation revealed that arginine administration prevents thymic involution after surgery and increases lymphocyte counts [27, 28]. Moreover, it also became clinically apparent that there is an arginine requirement for proper wound healing [28–30].

Pursuant to such observations of arginine’s immunostimulatory effects, dietary arginine supplementation gained attention, with the aim of creating so-called immune-enhancing diets (IED) [31–33]. These diets included between two and six times greater arginine contents than normal diets, as well as *n*-3 fatty acids, nucleotides, and other micronutrients with alleged immunostimulatory effects. Numerous IED trials conducted in a variety of patient populations have unfortunately resulted in mixed outcomes [32]. For instance, high-risk surgery patients benefited from IED diets by exhibiting reduced infections, and trauma patients exhibited an increase in markers of enhanced immunity [34–36]. Conversely, IED diets failed to show any benefit in critically ill non-surgical patients, with controversial effects in sepsis patients [31–33, 37], for which arginine supplementation remains of questionable value or is even counter-indicated [38, 39]. Determining whether arginine metabolism regulates immune cell functions in specific diseases will undoubtably foster the development of personalized therapeutic interventions in pathologies, where modulating arginine metabolism proves to be beneficial.

## Dietary arginine intake and organismal-level arginine synthesis

The turnover of circulating arginine in human adults is rapid, with half-life measurements ranging from 41.6 to 79.5 min depending on the administration route and arginine dose [40]. The plasma arginine concentration ranges from 6.7 to 81.6 μmol/L in young humans and from 7.8 to 113.7 μmol/L in the elderly [41, 42], and five main factors regulate its plasmatic concentration: dietary arginine intake, endogenous arginine synthesis, arginine catabolism, hepatic urea excretion, and protein turnover. Although both the diet and biosynthesis significantly contribute to arginine fluxes, protein turnover remains the major contributor to the circulating pool of free arginine [43]. Modern western diets provide, on average, a daily intake of 3–6 g of arginine [44, 45]. Orally ingested arginine is absorbed in the jejunum and in the ileum via a specific amino acid transporter, classically known as the y^+^ transporter, which mediates uptake of the basic amino acids lysine, ornithine, histidine, and arginine [46, 47]. In human adults, however, arginase activity in the intestinal mucosa degrades approximately 40% of the arginine absorbed in the small intestine [48].

The endogenous synthesis of arginine yields a daily production of 15–20 g of arginine [49]. However, arginine dietary supply does not alter its synthesis rates, and increased arginine synthesis is not used to counterbalance a reduction in dietary intake [12]. Although hepatocytes synthesize arginine efficiently, urea cycle enzymes are organized in a metabolon that channels de novo synthesized arginine to arginase-mediated hydrolysis [50, 51], thus resulting in little to no net synthesis of arginine in the liver. Furthermore, only about 5% of the urea produced by the liver derives from plasma arginine [52]. The liver is, therefore, regarded as an arginine-depleted organ, in which arginine concentrations (0.03–0.10 μmol/L) are considerably lower than those of other amino acids (0.5–10.0 μmol/L) [53]. This also reflects strict segregation between hepatic and circulating arginine pools.

**Fig. 1:**
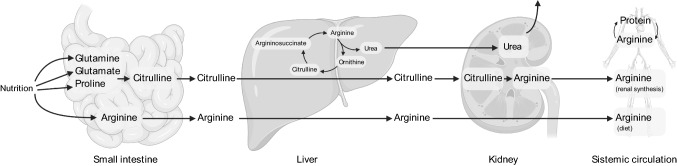
Inter-organ arginine metabolism and the intestinal renal arginine synthesis axis

Unlike arginine synthesis in the urea cycle, most de novo arginine synthesis entails an inter-organ pathway: the intestinal–renal axis [54, 55] (Fig. 1). In a first step, enteral glutamine and glutamate, as well as circulating glutamine, are taken up by the small intestine and finally converted to citrulline in a series of reactions initiated by l-∆^1^-pyrroline-5-carboxylate (P5C) synthetase. Citrulline is subsequently released by the intestines into the bloodstream, with an efficient bypass of the first-pass metabolism. Once in the systemic circulation, virtually, all fluxes of citrulline in the plasma feed into de novo arginine synthesis by the kidneys, being up taken at the proximal tubule of the nephron [56–58]. In the kidney, argininosuccinate synthase (ASS1) converts citrulline to argininosuccinate, and subsequently, argininosuccinate lyase (ASL) converts it to arginine (Fig. 1), which ultimately reenters circulation in the bloodstream. The kidney is, therefore, the primary organ responsible for maintaining circulating arginine levels [54, 55]. In certain conditions, macrophages can also regenerate arginine pools thanks to the reconversion of citrulline to arginine via ASS1 and ASL [59, 60]. Importantly, however, not all cell types concomitantly express all the enzymes required for de novo arginine synthesis; hence, certain cell types, notably cells of the immune system, depend on circulating supplies of arginine or its immediate precursors.

## Intracellular arginine metabolism

### Cellular uptake

Intracellular arginine concentrations are considerably greater than those in the extracellular microenvironment or in the blood. Plasma arginine concentrations in healthy adults oscillate between 0.05 and 0.20 mmol/L, while intracellular arginine concentrations range from 0.10 up to 1.00 mmol/L depending on the cell type [61–63]. Lipid membranes are not permeable to the positively charged arginine molecule, which prevents its free diffusion and permits significant compartmentalization of different arginine pools [64]. The build-up of intracellular arginine pools, therefore, entails the involvement of transporter systems.

Cellular uptake of arginine involves several different amino acid transport systems, including mainly the cationic amino acid transporters CAT-1 and CAT-2 [65, 66], with minor involvement of the LAT-1 and LAT-2 proteins and the B^o^, + and b^o^, + systems [67, 68]. The principal arginine transporter in most cell types is CAT-1, which translocates arginine in a Na^+^-independent manner and can be *trans*-stimulated by other cationic amino acids [69]. Different peripheral cells express CAT-1, such as macrophages, platelets, endothelial cells, and vascular smooth muscle cells. The CAT-2 transporter exists in two isoforms: CAT-2A and CAT-2B, of which only the latter exhibits high affinity for arginine and is expressed in macrophages and T cells [68, 70]. Notably, the CAT-1 transporter can be competitively inhibited by lysine, ornithine and canavanine, as well as by certain inhibitors of the arginine-metabolizing enzyme nitric oxide synthase 2 (NOS2), such as l-NMMA and l-NIO [71–73]. Therefore, certain NOS2-inhibiting pharmacologic interventions can interfere with the availability of arginine for other arginine-metabolizing enzymes.

In several biological systems, the induction of arginase and NOS2 enzymes is concomitant with the upregulation of arginine transporter expression [74–76]. Moreover, cytokines, such as interferon gamma (IFN-γ) and tumor necrosis factor α (TNF-α), stimulate the activity of both arginine transporters and arginine-metabolizing enzymes [77, 78]. These observations suggest that these proteins constitute a functional unit of the response to metabolic demands imposed by immune challenges.

Although arginine transporters in the plasma membrane are well characterized, knowledge about the transport of arginine through mitochondrial membranes remains quite limited. Recent reports demonstrate that the SLC25A29 gene encodes a mitochondrial transporter of basic amino acids, amongst them arginine [79]. However, mechanisms mediating arginine trafficking into the mitochondria of immune cells remain unexplored.

### Catabolism

The largest fraction of the circulating free-arginine flux is expended for protein synthesis —via loading of arginine onto arginyl-tRNAs by arginyl-tRNA synthetases [80]. Arginyl-tRNAs also catalyze the conjugation of arginine to the N-termini of proteins and their consequent degradation by the ubiquitin-dependent proteolytic pathway [81]. Strikingly, ammonia detoxification only consumes a very minor portion of the arginine flux. Besides protein turnover and ammonia detoxification, a number of distinct metabolic pathways catabolize the remainder proportion of circulating arginine, thus serving different functions which, frequently, are concomitantly active in the same cell.

#### Arginases

**Fig. 2:**
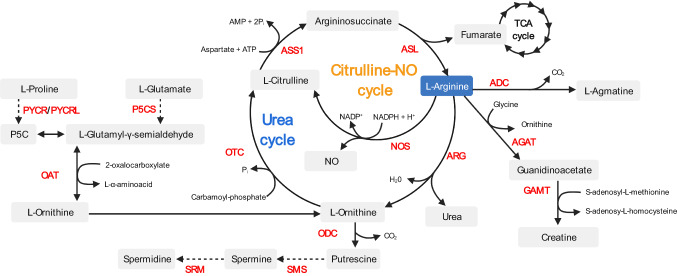
Overview of pathways involved in arginine metabolism. Abbreviations: *ADC* arginine decarboxylase, *AGAT* arginine:glycine amidinotransferase, *GAMT* guanidinoacetate *N*-methyltransferase *ARG* arginase, *NOS* nitric oxide synthase, *ASL* argininosuccinate lyase, *ODC* ornithine decarboxylase, *ASS1* argininosuccinate synthase 1, *OTC* ornithine carbamoyltransferase, *SMS* spermine synthase, *SRM* spermidine synthase, *P5CS* delta–1–pyrroline–5–carboxylate synthase, *PYCR* pyrroline–5–carboxylate reductase, *PYCRL* pyrroline–5–carboxylate reductase-like, *OAT* ornithine aminotransferase

The arginase pathway leads to arginine hydrolysis and the consequent production of urea and ornithine. Urea, a toxic water-soluble metabolite, is efficiently excreted by the kidneys. Ornithine can be further metabolized by ornithine aminotransferase (OAT) and other enzymes to generate proline or glutamate or, alternatively, by ornithine decarboxylase (ODC) to generate putrescine and downstream polyamine metabolites [1, 43] (Fig. 2). Another prominent fate of arginase-derived ornithine is to feed the urea cycle: ornithine can be subsequently converted to citrulline by ornithine carbamoyltransferase (ODC) and, via arginine-synthesizing enzymes ASS1 and ASL, citrulline is used to regenerate arginine and hence iterate the next cycle of the urea cycle (Fig. 2).

Mammals express two arginase isoforms (Arg1 and Arg2) encoded by different genes. Although there is strict conservation of the catalytic site [82], the human isoforms exhibit only 58% amino acid homology. The isoforms differ in their tissue distribution and subcellular localization. Whereas Arg1 does not contain any targeting sequence, and is thus located in the cytosol, both human and mouse Arg2 have an *N*-terminal mitochondrial targeting sequence that is cleaved during protein maturation [82, 83]. In ureotelic organisms, only the Arg1 isoform is highly expressed in the liver, where it ensures that the arginase activity required for the urea cycle. Outside of the liver, Arg1 expression circumscribes to discrete cell types and is under transcriptional control by cytokines. On the contrary, Arg2 presents a more ubiquitous and constitutive expression pattern, independent of regulation by cytokines. Phylogenetic analysis suggest that Arg2 is the ancestral gene from which Arg1 arose via a duplication event during the evolution of vertebrates to terrestrial adaptation and the emergence of a need for water-soluble excretion of nitrogen [84].

#### Nitric oxide synthases

Nitric oxide synthases (NOS) catabolize arginine, resulting in the generation of nitric oxide (NO) and citrulline. Notably, arginine is the sole substrate used for NO synthesis [85]. Mammals have three distinct NOS isoforms (NOS1, NOS2, and NOS3) encoded by different genes. All three isoforms require BH_4_, haem, FAD, and FMN as cofactors for maximal activity, but differ with respect to their intracellular localization, kinetic properties, inhibitor sensitivity, and expression patterns. NOS1 and NOS3 are constitutively expressed in neuronal and endothelial cells, respectively. In endothelial cells, NO serves as a signaling molecule for inducing vasodilation: endothelium-derived NO regulates smooth muscle diastole, thereby increasing blood flow. It also inhibits platelet aggregation [86] and leukocyte aggregation [87]. NOS2 is the prevalent isoform in immune cells, where it is not constitutively expressed, but is strongly induced by lipopolysaccharide (LPS) and inflammatory cytokines: it is hence known as inducible NOS or iNOS. Once expressed, NOS2 is constitutively active and its activity only depends on regulation by its substrate—arginine promotes dimerization of catalytically active NOS2 dimers—or cofactor availability [88].

NOS2 is most often implicated in the production of NO during pro-inflammatory responses. NO can react with other oxygen species to generate nitrite and nitrate as well as other highly cytotoxic reactive nitrogen species (RNS). The biological roles of NO are multiple and its synthesis during inflammatory conditions can entail both beneficial, e.g., liver protection in LPS-treated rats [89], and detrimental consequences, e.g., aggravated ulcerative colitis and mucosal destruction [90]. NO is also a potent cytostatic and cytotoxic molecule that can inhibit cell growth or kill cells in an unspecific way. For instance, high local concentrations of NO can damage cells by condensing amino acid and/or thiol groups, activating the p53 pathway [91], or inducing cell necrosis caused by the peroxynitrite-oxidizing radical [92]. Likewise, low NO concentrations can also impair specific cellular functions. For example, Complex IV of the respiratory electron transport chain is highly sensitive to NO inhibition [93]. Therefore, the complex and diverse consequences of NO synthesis require regulatory mechanisms that unleash NOS2 activity only under precise conditions.

Paradoxically, NO synthesis rates depend on arginine concentration, even though the low *K*_m_ of NOS enzymes implies that these enzymes should be saturated at all physiological arginine concentrations [94]. This irreconciled observation is known as “the arginine paradox” and numerous theories have aimed at disentangling this conundrum, such as the existence of non-interchangeable intracellular pools of arginine [64] or general control nonderepressible 2 (GCN-2)-mediated transcriptional control of NOS2 by arginine availability [95]. An additional mechanism regulating NOS activity involves substrate competition with arginase enzymes: although *K*_m_ of ARG1 and ARG2 are 3000-fold higher than that of NOS2, *V*_max_ of arginases are 1000-fold greater than that of NOS2. Hence, arginases and NOS2 catabolize arginine at similar rates [96] and effectively compete with each other for their substrate when co-expressed [43, 97]. A number of additional mechanisms also contribute to the crosstalk between the arginase and NOS2 pathways. First, the intermediate subproduct *N*-ω-hydroxy-l-arginine of the reaction catalyzed by NOS2 is an inhibitor of arginase activity [98]. Second, arginine availability controls the translation of NOS2, which can, therefore, be modulated by arginase activity [95]. Last, arginine depletion by arginase promotes the uncoupling of NOS2, which impedes NO synthesis and promotes the generation of RNS (reviewed below).

#### Creatine pathway

In a similar vein as for NO production, arginine is the only amino acid that provides the amidino group for creatine synthesis [43]. In this pathway, arginine serves as a donor for the transfer of the amidino group to a glycine backbone. l-arginine:glycine amidinotransferases (AGAT) catalyze this reaction, to generate ornithine and guanidinoacetic acid. The latter is subsequently methylated by *S*-adenosyl-l-methionine:*N*-guanidinoacetate methyltransferases (GAMT), to ultimately produce creatine and *S*-adenosylhomocysteine. Although systemic creatine synthesis involves an inter-organ pathway [7, 99], the expression of creatine-synthesizing AGAT and GAMT activity has been documented in human and rat spleens, respectively [99]. Of note, the creatine kinase enzyme can reversibly phosphorylate creatine to phosphocreatine, using adenosine triphosphate (ATP) as phosphate donor or acceptor. Creatine thus serves as major energy storage and transport molecule, having major implications for the energy-demanding biosynthetic processes performed by immune cells.

#### Agmatine and polyamine pathways

Plants and bacteria—including human microbiota species—express arginine decarboxylase (ADC) enzymes that catabolize arginine to agmatine and CO_2_. The presence of an ADC gene in humans is controversial [100], although agmatine synthesis has been detected in the small intestine, brain, kidney, liver and adrenal tissue, as well as in macrophages [101–104]. However, the direct effects of agmatine on immune cells remain unstudied, and it remains unclear whether immune cells synthesize agmatine via arginine catabolism or they import it from exogenous sources, such as the diet and microbiota synthesis. Nevertheless, agmatine catabolism converges with the arginase pathway, as it results in the synthesis of ornithine. The resulting ornithine can be further catabolized to generate different polyamine metabolites, which are well-known stimulators of cell growth and differentiation [1, 43].

## Arginine metabolism in immune cells

### Macrophages

Canonical classifications divide activated macrophages into two functional subsets: M1 or classically activated and M2 or alternatively activated [105, 106]. These two subsets catabolize arginine in a divergent manner—via predominant NOS2 or ARG1 activity, respectively—and the functional consequences associated with the dominant pathway are typical classifiers for each subset [1, 107, 108]. However, basing the classification of activated macrophages on the polarization of arginine metabolism is an oversimplification of the complex immunobiology of activated macrophages, as they may express neither ARG1 nor NOS2 [109], or use both pathways concomitantly, as observed in murine macrophages after LPS stimulation [110]. The expression of Arg2 in macrophages may also affect arginine catabolism [111, 112]. However, the importance of this contribution is ill-defined, as ARG2 expression is independent of cytokines that affect macrophage function and ARG2 is not the predominantly active arginase in these cells [113, 114]. The roles of creatine and agmatine synthesis in macrophages also remain poorly defined. However, recent studies indicate that the uptake of creatine in macrophages promotes IL-4 and STAT6 induces ARG1 expression, and the suppression of M1 polarisation [115]. Similarly, agmatine promotes ARG1 expression in vivo [116] and suppression of the M1 phenotype in vitro [117, 118].

#### M1 or classically activated macrophages

M1 macrophages predominantly catabolize arginine via NOS2. Th1 cytokines drive M1 activation and induce NOS2 activity while inhibiting Th2-driven ARG1 expression [1]. The synthesis of NO endows M1 macrophages with pro-inflammatory and microbicidal properties, and renders them proficient for cytotoxic clearing of intracellular pathogens and malignant cells [119–122]. In these macrophages, Th1 inflammatory cytokines, such as IFN-γ, IFN-α, IFN-β, and IL-1, drive the activation of transcription factors, such as NF-*κ*B, AP-1, IRF1, and STAT1 [123–126]. The transcriptional program activated by these factors induces NOS2 expression [127] and co-expression of arginine transporters [71, 128] and enzymes implicated in the synthesis of NOS2 cofactors [129–132]. However, intense NOS2 activity could limit arginine availability and, therefore, impair NO synthesis. To circumvent arginine scarcity, citrulline can replenish the intracellular arginine pool, as stimuli, such as IFN-γ and Toll-like receptor (TLR), agonists also induce ASS1 expression. ASS1, coupled to constitutively expressed ASL, allows the recycling of citrulline into argininosuccinate and subsequent de novo arginine synthesis. The importance of citrulline recycling—known as the citrulline-NO cycle—is manifested in Ass1^−/−^ macrophages, which fail to control mycobacterial infections [133].

#### M2 or alternatively activated macrophages

M2 macrophages predominantly catabolize arginine via ARG1. In this instance, the Th2 cytokines IL-4 and IL-13 promote Arg1 upregulation via binding of STAT6, together with STAT3 and C/EBP*β*, to an enhancer in the Arg1 locus [134–136]. Likewise, Arg1 expression can also be induced via autocrine cytokines produced by mycobacteria-infected macrophages, such as IL-10, IL-6, and GM-CSF [137], as well as by other soluble factors, such as TGF-β, PGE2, catecholamines, cAMP, and TLR agonists [138–140]. The functions of M2 macrophages are, in part, mediated by the induction of ornithine, proline and polyamines synthesis [141, 142]; pathways that enable mechanisms essential for regulating humoral immunity, anti-parasitic responses, allergy, and fibrosis and wound repair processes [143]. For instance, in vitro pharmacologic inhibition of Arg1 and macrophage-specific deletion of Arg1 have indicated that Arg1-expressing macrophages are critical for matrix deposition and wound healing [144]. A potential explanation of such effects is that arginase activity potentiates collagen production via increased proline synthesis [145, 146]. In line with this notion, the metabolism of arginine in wounds presents a biphasic pattern: a first early burst of microbicidal NO synthesis precedes a drop in arginine concentration and an increase in ornithine and proline synthesis to promote wound healing [146, 147]. In addition, Arg1-mediated wound healing mechanisms can be employed to directly modulate parasite growth. Using an unconventional mechanism, antibodies against *Heligmosomoides polygyrus* larvae trigger M2/alternative activation of macrophages recruited to infection sites, and subsequently, Arg1 activity from M2 macrophages generates ornithine and polyamines that decrease larvae motility and prevent tissue damage caused by the helminth [148].

#### Arginases and NOS2 crosstalk

As NO is a potent inflammatory and cytotoxic mediator, its uncontrolled synthesis may lead to collateral tissue damage. Macrophages, therefore, exploit the arginase pathway to competitively regulate NO production and thereby counterbalance exacerbated immunity. At the molecular level, the depletion of arginine alone can regulate NO production via GCN2-driven inhibition of eIF2*α* and a subsequent halt in NOS2 mRNA translation [95]. In addition, spermine produced by the arginase pathway can reduce mRNA levels for NOS2 and the CAT-2B transporter, as well as suppress TLR-driven cytokine synthesis by rat macrophages [149, 150]. As exemplified by murine models of schistosoma mansoni infection, a deficiency in Arg1 in macrophages results in uncontrolled Th2 cytokine-driven liver inflammation and fibrosis [151], as well as intestinal inflammation caused by a dysregulated Th17/Treg ratio and the synthesis pro-inflammatory IL-6, IL-12/IL-23p40, and NO [152]. Notably, however, Arg1 activity can also coexist with NOS2 activity. During *Mycobacterium tuberculosis* infection, a particular organization of macrophages in granulomas leads to the concentration of NOS2 activity in the inner regions, whereas Arg1 activity predominates in the outer regions. This helps to minimize lung pathology by surrounding zones presenting microbicidal activity with regions favoring tissue repair [153]. Such protective roles of arginase activity are nonetheless pathology-specific, as host Arg1 is irrelevant in some models of excessive lung inflammation and asthma [143].

Pathogens can co-opt macrophage Arg1 activity to blunt NO synthesis and escape the host immune response. For instance, *M. tuberculosis* coinfections with either *Schistosoma mansoni* or *Toxoplasma gondii* lead to increased Arg1 activity in lung macrophages and exacerbated disease progression [154, 155]. Pathogens can also exploit arginase activities encoded in their own genomes. This situation is exemplified by *Helicobacter pylori*, which can blunt NOS2 activity in macrophages, thereby promoting pathogen survival, by expressing an arginase encoded by the rocF gene [156].

### Granulocytes: neutrophils

Although human granulocytes express arginase enzymes, their effector functions are surprisingly independent of arginine availability [157]. Instead, granulocytes can exert arginase-mediated immunosuppressive functions [158] reminiscent of myeloid-derived suppressor cells (MDSCs), a heterogeneous population of myeloid cells having paramount relevance for pathological disruption of arginine metabolism. Neutrophils are thus often included in the immunosuppressive MDSC population. Although neutrophils constitutively express Arg1, this enzyme is not used to metabolize arginine intracellularly [158, 159]. Instead, they are key regulators of extracellular arginine availability as they secrete arginase-containing granules [160], a mechanism having extensive pathological implications in several conditions. For instance, compared to neutrophils from healthy individuals, neutrophils from septic shock patients display elevated Arg2 expression and a superior capacity to limit T cell proliferation [161]. Pharmacological arginase inhibition confirmed that this suppressive capacity is indeed partially dependent on arginase activity.

On the other hand, arginine can also enhance the phagocytosis of *Staphylococcus aureus* by human neutrophils [162] and sustain the synthesis of NO by mouse neutrophils [163]. NO synthesis in neutrophils can also exert a protective role, in this case, by preventing the production of highly damaging superoxide by these cells [164, 165].

### Myeloid-derived suppressor cells

The MDSC population encompasses diverse types of developmentally immature cells at different stages of myelopoiesis [166]. They share the unifying property of being able to suppress T cell function. Although MDSCs are uniformly defined by the expression of CD11b, they exhibit considerable heterogeneity in terms of (1) their expression of arginine-catabolizing enzymes, (2) their pluripotency with respect to being able to differentiate into macrophages, granulocytes, or dendritic cells depending on the cytokine and growth factor culture conditions [167], and (3) the mechanisms they employ for exerting T cell inhibition. Arg1-expressing MDSCs are prominent immunosuppressors implicated in the pathogenesis of chronic helminth infection, autoimmune diseases and graft-versus-host disease, as well as in cancer [168–173]. In neoplastic diseases, MDSCs are major contributors to the intra-tumoral arginase activity observed in both preclinical models and cancer patients [167, 174–176], in which tumor-derived factors, such as PGE2, can induce Arg1 expression via STAT3 or STAT6 [167, 177, 178]. A classic immunosuppressive mechanism ascribed to MDSCs is depletion of the extracellular arginine pool by Arg1, resulting in a reduced availability of arginine for effector T cells [172]. Moreover, co-expression of CAT-2B in MDSCs further supports the arginine-depleting activity of Arg1 by importing more extracellular arginine in exchange for the newly synthesized ornithine [179–181].

Arginine depletion by MDSCs exerts immunosuppressive effects via two main mechanisms. First, suboptimal concentrations of arginine lead to decreased NOS2 mRNA translation, reduced NO synthesis, and induced NOS2 uncoupling [95, 182]. When arginine is scarce, the reductase and oxygenase domains of uncoupled NOS2 can only transfer NADPH electrons to O_2_, thereby generating O_2_^−^ superoxide and, via subsequent reactions, ROS and RNS [183, 184]. The production of these oxidizing species within arginine-starved T cells or by neighboring MDSCs affects numerous key processes [185–187], including impaired loading of antigenic peptides onto MHC complexes in tumor cells [188], reduced responsiveness of T cells to cytokines or to antigen-specific stimulation [189], and even active induction of T cell hyporesponsiveness [190]. In a second, complementary manner, arginine starvation has direct, cell-intrinsic effects on T cells, as described in more detail below. Although MDSC activity can induce pathogenic T cell suppression, for example, by inhibiting anti-tumor T cells, it can also exert beneficial effects by limiting unwanted inflammation [191]. For instance, both IFN-γ and IL-4 activate MDSCs at inflammation sites, thereby initiating a negative feedback loop that suppresses T cell activity and helps to resolve inflammation [74, 179].

### Dendritic cells

Dendritic cells (DCs) are critical relays between the innate and adaptive immune systems: they integrate multiple signals, for example cytokines secreted by innate immune cells [192, 193], to ultimately instruct either reactive or tolerant adaptive immune responses. Arginases and NOS2 enzymes are prominent regulators of arginine metabolism in DCs, and in a dichotomy reminiscent to that observed for macrophages, the differential use of these pathways dynamically modulates their stimulatory outcomes. Therefore, the expression of these enzymes is tightly controlled in DCs.

In the previous studies, we identified Arg2 as a target of microRNA-155 (miR-155) in diverse activated murine DC subsets [194]. We demonstrated that in contrast to macrophages stimulated with CpG and IFN-γ [195], Arg2 expression is silenced by miR-155 in activated DCs. In a series of in vitro and in vivo experiments, we demonstrated that the repression of Arg2 by miR-155 in DCs is a prerequisite for their ability to induce optimal T cell priming [196]. Consequently, de-repressed Arg2 activity in activated miR-155^−/−^ DCs thwarts cognate T cell activation and proliferation.

The relevance of arginine metabolism in DCs is further highlighted by the identification of a specific DC subpopulation that is characterized by TNF-α and NOS2 expression, hence known as TNF-α, iNOS-producing DCs (Tip-DCs) [197, 198]. Tip-DCs exert pro-inflammatory roles conferring resistance to *Listeria monocytogenes*, *Brucella melitensis,* and *Leishmania major* infections [198–200]. Besides pathogen resistance, the pro-inflammatory activity of Tip-DCs and their interaction with tumor-specific CD8^+^ T cells result in superior anti-tumor responses [201]. Conversely, Tip-DC-derived NO suppresses antigen-specific T cells and prevents the exacerbation of autoimmune myocarditis in vivo [202]. The activity of Tip-DCs also has detrimental consequences in *Trypanosoma brucei* rodent infection, a model in which IL-10 genetic administration counters pathological liver damage via the inhibition of Tip-DC maturation [203].

Dendritic cells can also acquire tolerogenic properties and exert immunosuppressive effects by modulating their metabolism of arginine. For instance, murine DCs can reinforce the suppressive activity of TGF-β-stimulated MDSCs: in response to ornithine and spermine synthesized via the arginase pathway in MDSCs, DCs upregulate Arg1 and indoleamine 2,3-dioxygenase 1 (IDO1), thereby further strengthening the immunosuppressive program triggered by TGF-β [204]. Another prominent example is fetal conventional DCs (cDCs). Fetal cDC subpopulations are similar to those present in adults, with one major exception: fetal cDCs promote tolerogenic responses and generate higher frequencies of FOXP3^+^ regulatory T cells (Treg). A recent report demonstrated that the co-culture of adult T cells with fetal or adult DCs resulted in similar levels of T cell proliferation, but that the fetal DCs induced a reduction in the synthesis of the pro-inflammatory cytokines IL-2 and TNF-α and an increase in the production of IL-4 by the co-cultured T cells [205]. A key differentiating factor of the fetal DCs was a markedly greater expression of ARG2. The authors further showed that TNF-α expression was partially inhibited by ARG2-mediated arginine depletion, as arginine supplementation or pharmacological arginase inhibition restored TNF-α synthesis. These observations emphasize the importance of arginine depletion as a physiological strategy implemented by DCs to prevent detrimental T cell reactivity.

### Erythroid cells

Erythroid cells also express arginine-metabolizing enzymes [206, 207]. Using analogous mechanism to fetal DCs, ARG2 activity in CD71^+^ erythroid cells contributes to the suppression of innate and adaptive immunity in immunologically delicate contexts, such as during pregnancy or postnatally. Interestingly, pregnancy induces a subset of maternal CD71^+^ erythroid cells that suppress allogeneic T cell responses via ARG2 activity and PD-L1 expression, therefore, promoting fetomaternal tolerance [208]. However, ARG2 is expressed not only in maternal CD71^+^ erythroid cells, but also in neonatal CD71^+^ erythroid cells. Therefore, neonates actively engage arginine-depleting immunosuppressive mechanisms, in opposition to the established notion that immaturity of immune cells underlies suboptimal immunity in neonates [209]. In line with this notion, recent investigations demonstrated that CD71^+^ erythroid cells suppress both cellular and humoral adaptive immune responses against *Bordetella pertussis*—a common pathogen of the neonatal respiratory tract—as well as innate immune responses [210, 211]. More specifically, Arg2-expressing CD71^+^ erythroid cells regulate CD11b^+^ cell phagocytosis in an arginine-dependent manner. They also illustrate how ancient metabolic functions, such as mitochondrial arginase activity, can be repurposed to modulate appropriate immune reactivity in immunologically delicate scenarios, such as to maintain fetomaternal tolerance by preventing undesirable immune reactivity directed against the fetus.

### T cells

A seminal study in 1968 first demonstrated that arginine depletion in T cell lymphocytes infected by arginase-expressing mycoplasma inhibits their activation and proliferation in vitro [25]; this inhibition was arginine-dependent as arginine repletion at 10 mol/L relieved the block in activation and proliferation. Subsequent experiments confirmed that arginine depletion depresses T cell proliferation in vitro in a dose-dependent manner, and that maximal proliferation occurred after replenishing arginine to the concentrations typically found in plasma (around 100 μmol/L) [82, 212, 213]. Further increases in arginine concentrations beyond this threshold had no additional effect on proliferation [5, 214, 215]. Notably, proliferation and memory formation presented a more marked dependency on arginine availability for CD8^+^ T cells than for CD4^+^ T cells [216]. In addition, dietary arginine supplementation improved thymic weight and thymic lymphocyte counts in rats, increased ex vivo reactivity of human and rat T lymphocytes to PHA and concanavalin A [30, 217], and T cell cytotoxicity [218].

#### Effects of arginine starvation on T cell biology

**Fig. 3:**
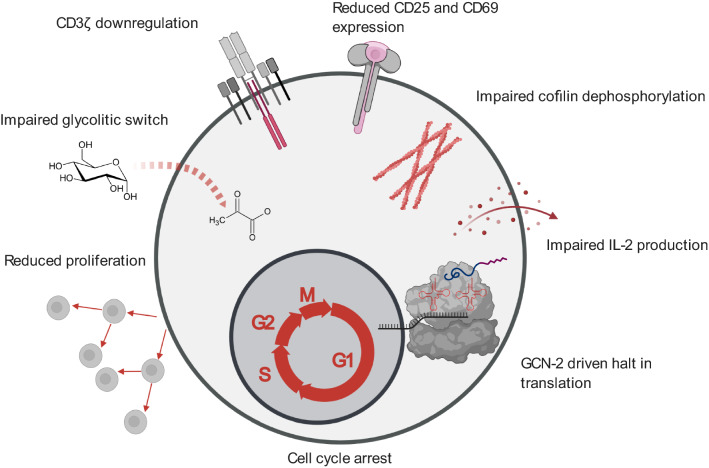
Overview of the deleterious effects that arginine starvation exerts on T cell functions

A plethora of studies has demonstrated that the culture of T cells under limiting arginine concentrations impairs their function via downregulation of the CD3ζ subunit of the T cell receptor (TCR) complex (Fig. 3). This subunit is indispensable for the assembly of the TCR complex [219–221] and couples TCR ligation with the downstream signal transduction cascade mediating T cell activation via phosphorylation of ITAM motifs on the CD3ζ subunit. Remarkably, ex vivo arginine availability within physiological ranges modulates the expression level of CD3ζ [222, 223] via a mechanism that is not yet fully elucidated [224]. Interestingly, supplementation of culture media with citrulline, a precursor of arginine synthesis, can increase CD3ζ expression under low arginine conditions by extending the half-life of its mRNA [223]. Downregulation of CD3ζ expression blocks T cell proliferation without impairing T cell viability [158] (Fig. 3). Collectively, these findings indicate that regulation of CD3ζ expression is a prominent mechanism for modulating T cell activation. Several examples underline the physiological relevance of these findings. For example, as mentioned above, *H. pylori* co-opts this regulatory mechanism to inhibit CD3ζ expression and thereby restrict T cell activity by depleting arginine via the arginase enzyme encoded by its rocF gene [225]. Other studies have demonstrated that dying polymorphonuclear leukocytes release Arg1 and thereby induce the depletion of extracellular arginine, leading to the loss of CD3ζ expression by T cells in inflamed microenvironments [158, 179].

In addition to CD3ζ downregulation, arginine deprivation modulates TCR signaling by impairing cofilin dephosphorylation in activated human T cells [226] (Fig. 3). Dephosphorylated cofilin increases the dynamics of F-actin networks [227], which is critical for T cell activation, because it regulates TCR sensitivity thresholds in immune synapses [228]. Impaired cofilin dephosphorylation correlates with a decrease in the F-actin content and decreased accumulation of CD2 and CD3 in the immune synapse of activated T cells, ultimately decreasing T cell proliferation and cytokine synthesis [226]. In vitro activation with phorbol myristate acetate (PMA), which bypasses the requirement for TCR signaling, does not suffice to rescue poor T cell proliferation in arginine-depleted conditions [229]. This observation implies that the inhibitory effect of arginine depletion on T cell proliferation is not caused exclusively by decreased CD3ζ expression and TCR signaling. Other mechanisms must also be at play. In agreement with this, arginine depletion has been implicated in other molecular alterations, such as impaired nuclear translocation of NF*κ*B-p65 [229].

Arginine deficiency leads to decreased IL-2 production by cultured human T cells, and reduced expression of the early activation markers CD25 and CD69 [216] (Fig. 3). Seminal publications first proposed that arginine deprivation decreases IL-2 concentrations in T cell culture supernatants by regulating translation of its mRNA and by modifying its autologous consumption. However, minimal arginine concentrations of 10–100 μM suffice for enabling robust T cell proliferation and IL-2 production [216].

Arginine starvation also regulates d-type cyclins (D1, D2, and D3) and cyclin-dependent kinases (CDK4 and CDK6) in T cells, therefore, regulating progression through the cell cycle [230] (Fig. 3). For instance, in activated T cells, the absence of arginine blocks cyclin D3 and CDK4 upregulation, but increases CDK6 expression and results in the arrest of T cells in the G_0_–G_1_ phase of the cell cycle [231, 232], suggesting that sufficient arginine is a requirement for cell cycle entry.

Amino acid depletion halts protein synthesis due to the accumulation of uncharged aminoacyl-tRNAs, leading to activation of the kinase GCN2, which senses the binding of uncharged tRNAs to ribosomes and halts mRNA translation. More precisely, activated GCN2 phosphorylates the translation initiation factor eIF2*α* and inhibits protein synthesis by blocking the binding of the eIF2 complex to methionine aminoacyl-tRNA [233]. GCN2 activation leads to an arrest in T cell proliferation, induces T cell anergy, impairs cytotoxic effector functions, and further downregulates the expression of CD3ζ in mouse CD8^+^ T cells [234, 235]. In line with these observations, the absence of arginine promotes the phosphorylation of eIF2*α* and is associated with decreased translation rates [236] (Fig. 3).

A recent report demonstrated that arginine starvation can also impact the glycolytic switch in activated T cells: arginine starvation caused by adding recombinant Arg1 to cultured T cells blocked their glycolytic function, without affecting mitochondrial biogenesis or mitochondrial functionality [237] (Fig. 3). Conversely, the addition of supraphysiological concentrations of arginine to T cell cultures resulted in perturbations of energy metabolism and promoted CD8^+^ T cell anti-tumor activity in vivo upon reinfusion into tumor-bearing mice [5]. Remarkably, T cells also employ mechanisms to sustain intracellular arginine pools and counteract arginine deprivation: when confronted with arginine scarcity caused by, for instance, Arg1-expressing myeloid cells [172], Jurkat T cells upregulate the transcription of the ASS1 gene to promote arginine biosynthesis [218]. In line with this, reengineering CAR-T cells to express arginine-synthetizing ASS1 and OTC enhances CAR-T cell in vivo persistence and activity against solid and hematologic tumors [238].

#### Arginase 2 regulates the intracellular metabolism of arginine in T cells

**Fig. 4:**
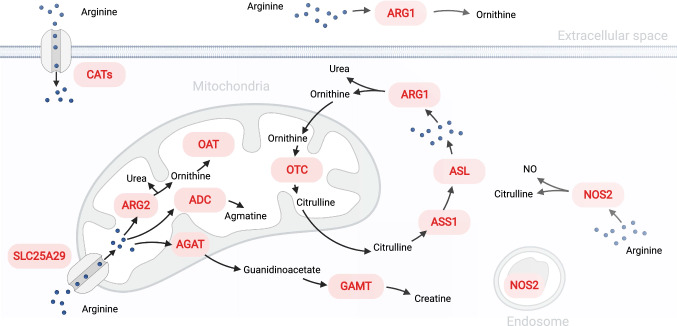
Subcellular localization of proteins involved in arginine metabolism. Abbreviations: *ADC* arginine decarboxylase, *AGAT* arginine:glycine amidinotransferase, *ARG* arginase, *NOS2* nitric oxide synthase 2, *ASS1* argininosuccinate synthase 1, *ASL* argininosuccinate lyase, *CATs* cationic amino acid transporters, *GAMT* guanidinoacetate *N*-methyltransferase, *OTC* ornithine carbamoyltransferase, *OAT* ornithine aminotransferase

Most investigations on the influence of arginine metabolism on T cells have focused on mechanisms affecting extracellular arginine concentration, such as Arg1-mediated arginine depletion in the tumor microenvironment. Nonetheless, T cell activation encompasses an intense arginine uptake [4, 5], and in human endothelial cells, distinct intracellular arginine pools are not freely interchangeable [64]. Hence, the extracellular arginine supply may not serve as a reliable proxy for intracellular arginine availability. Mounting evidence suggests that the intracellular metabolism of arginine profoundly alters T cell function. Arg2 has recently emerged as a cell-autonomous regulator of mouse and human activated T cells. Notably, pharmacological inhibition of arginases increases in vitro activation and survival of human T cells, which express ARG2 but not ARG1 [3]. Along similar lines, activated Arg2^−/−^ mouse T cells present enhanced in vitro survival and increased intracellular arginine levels—the latter being indicative of reduced arginase activity in these cells [3]. Importantly, such experiments were performed under excess arginine conditions, hence independent of extracellular arginine availability, and, therefore, argue for a cell-intrinsic role of the mitochondrial Arg2 (Fig. 4) in the reshaping of T cell arginine metabolism.

In a subsequent study, we demonstrated that germ-line Arg2 deletion and adoptive transfer of Arg2^−/−^ CD8^+^ T cells significantly reduce tumor growth in preclinical cancer models by enhancing CD8^+^ T cell activation, and endowing these cells with more robust cytotoxic function, memory T cell formation, and persistence [215]. Furthermore, Arg2-deficiency in CD8^+^ T cells strongly synergized with PD-1 blockade for the control of tumor growth and animal survival. Although Arg2^−/−^ CD8^+^ T cells exhibited accelerated and more robust activation kinetics, their proliferative capacity remained unaffected. All these alterations occurred in an otherwise Arg2-proficient background in vivo and were independent of the extracellular arginine concentration in vitro. This suggests that the observed alterations were caused primarily by cell-autonomous mechanisms and emphasizes the critical importance of intracellular arginine pools relative to the extracellular arginine supply (Fig. 4).

Strikingly, we also observed that the depletion of CD4^+^ T cells selectively improves tumor control in Arg2-deficient mice. These results suggest a supportive role of Arg2 for the regulatory activity exerted by certain CD4^+^ T cell subsets in tumor-bearing mice. Interestingly, a very recent report demonstrated that T_regs_ found in healthy human skin and in metastatic melanoma [239] express Arg2. Conversely, the expression of Arg2 in T_regs_ infiltrating psoriatic skin is significantly less. Interestingly, the expression of Arg2 in T_regs_ attenuated mTOR activity and endowed them with enhanced suppressive activity in vitro and increased tissue persistence of adoptively-transferred T_regs_ [239]. Taken together, these observations suggest that the intracellular arginine concentration and its regulation by Arg2 dictate T_reg_ expansion and survival in different microenvironments, and unveil Arg2 as a putative therapeutic target in both autoimmune and neoplastic diseases.

#### Nitric oxide synthesis is a key modulator of T cell biology

Several studies have demonstrated that the NOS pathway can affect T cell biology. High NO concentrations can exert pro-apoptotic effects via different mechanisms, such as p53 accumulation and CD95 signaling [91, 240]. T cell activation also results in NO accumulation [241], in a mechanism regarded as a self‐limiting circuit that triggers T cell apoptosis via inhibition of the anti-apoptotic Bcl‐2 [242]. In line with these observations, endogenous NOS2 in mouse T cells regulates their contraction phase, endowing Nos2^−/−^ T cells with enhanced resistance to death by neglect and death after withdrawal of trophic stimuli [243]. Thus, mice lacking the Nos2 gene showed an increased frequency of CD4^+^ and CD8^+^ memory T cells after immunisation [243]. NO also dampens T cell proliferation and differentiation by impairing IL-2 synthesis [244, 245] and IL-2R signaling pathways [246]. NO can inhibit the binding of JAK3 to the common gamma-chain, thus dampening responses induced by the cytokine receptors for IL-2, IL-4, IL-7, IL-9, and IL-15 [247]. NO can also inhibit signaling via STAT5, a key factor for the maintenance of effector CD8^+^ T cell responses, as well as the ERK and AKT pathways [169]. In addition, RNOS derived from NO can affect conformational flexibility of binding of the TCR/CD8 complex to the pMHC-I (peptide-MHC-I) complex, thus limiting CD8 T cell anti-tumor activity [188, 248].

#### Creatine uptake supports anti-tumor T cell functions

Creatine and phosphocreatine buffer intracellular high-energy phosphate, acting as storage systems of the energy required for ATP synthesis [249]. The uptake of creatine has recently been reported to support anti-tumor T cell functions in murine melanoma and colorectal carcinoma models [250]. Tumor-infiltrating T cells express low levels of creatine-synthesizing enzymes AGAT and GAMT and primarily rely on creatine uptake via increased expression of its plasma membrane transporter SLC6A8 [250]. Interestingly, intraperitoneal creatine supplementation synergized with PD-1 blockade therapies for the control of tumor growth. These observations are reminiscent of similar synergistic results observed after PD-1 axis blockade in combination with dietary arginine supplementation [251] or Arg2 genetic deletion [215]. Therefore, whether arginine availability and its intracellular metabolism impact on creatinine levels in human T cells (see Fig. 2) is a question that merits further exploration.

### B cells

Only a few studies have addressed how arginine availability and arginine metabolism influence B lymphocyte biology. In comparison to T cells, B lymphocytes are less sensitive to arginine depletion. Decreased plasma arginine concentrations—via Arg1 overexpression in the small intestine—did not alter peripheral B cell proliferation or cytokine secretion. However, the same study demonstrated that arginine depletion led to an impaired developmental transition from pro-B to pre-B cells in the bone marrow, resulting in reduced B cell cellularity in secondary lymphoid organs [252]. In addition, mice fed with arginine-free diets showed impaired mucosal immunity against tetanus toxoid, as no toxin-specific fecal IgA were detected in these mice [253]. Single-cell RNA-seq experiments demonstrated that B cells in human lung tumors express ARG1 [254]. However, the roles of arginases in B cells remain mostly unaddressed. Conversely, it has been demonstrated that NOS2 is an intrinsic factor for plasma cell survival in vitro and in vivo*,* as its activity is critical for the response of plasma cells to pro-survival signals, provided by APRIL and IL-6 derived from bone-marrow stromal cells [255].

### Natural killer cells

The modulatory role of arginine extends to human natural killer (NK) cells. Early studies showed that arginine supplementation enhanced the cytotoxic activity of CD56^+^ human NK cells in vitro, as well as in vivo after 3 days of arginine supplementation in both healthy volunteers [256] and breast cancer patients [257]. Arginine supplementation in mice resulted in a similar observation, namely, enhanced poly IC-inducible NK cell activity [258]. Conversely, arginine starvation of cultured NK-92 cells and ex vivo human NK cells limited their proliferation, reduced expression of the NKp46 and NKp30 activating receptors, and of the NK *ζ* chain, and reduced their cytotoxicity and IFN-γ secretion [259]. Similarly, other studies showed that arginine depletion by arginase activity derived from human granulocytes severely impairs the proliferation and IFN-γ secretion by primary and in vitro IL-2-activated human NK cells [260]. Interestingly, these effects occurred by a GCN-2 independent mechanism. Furthermore, ARG1 activity derived from hepatitis C virus-induced MDSCs has also been shown to suppress IFN-γ production by NK cells [261].

Several reports have shown that the NOS pathway is active in NK cells. Unlike other immune cells, NK cells constitutively express the endothelial NOS or NOS3 [262]. The synthesis of NO by NK cells supports their cytotoxic functions in different in vitro settings. NK-derived NO synthesis has been proposed to act as an accessory cytotoxic mechanism that contributes to DNA fragmentation and cell lysis of NK targets [263]. In line with this hypothesis, a progressive increase in NO synthesis by IL-2 activated NK cells correlated positively with their capacity to lyse NK-resistant target cells [264]. Furthermore, the NOS inhibitors l-NMMA and l-NAME were able to partially inhibit the cytotoxic efficacy of NK cells [263, 264]. As mentioned above for granulocytes, NOS inhibitors can also decrease arginine-dependent NK cytotoxicity by interfering with arginine uptake.

## Arginine metabolism in cancer

Multiple reports have documented increased arginase activity in both animal models of cancer [167] and patients with colon, lung, breast, thyroid, or prostate cancer [174, 175]. Elevated arginine catabolism is a common hallmark of the TME. A pioneering study showed increased expression of arginases and NOS enzymes in neoplastic prostate epithelium compared to the surrounding healthy tissue, and that CD8^+^ T cells infiltrating these malignant tissues displayed a terminally differentiated phenotype, becoming irresponsive to immune stimuli [175]. In addition, treatment with arginase and NOS inhibitors restored CD8^+^ T cell responsiveness. Subsequent studies showed that Arg2 expression in mouse renal cell carcinoma suffices to induce arginine depletion and immunosuppress co-cultured human T cells [265]. These observations suggested, for the first time, that arginine depletion by arginases constitutes a major mechanism by which cancer cells induce in situ immunosuppression.

Enforcing arginine depletion in the tumor microenvironment might, however, seems counterproductive for tumor growth: amino acids are essential for tumor cell proliferation as well as for stromal and vascular tissue remodeling required for tumor spread. Several lines of evidence support the notion that immunosuppression by arginine depletion is more beneficial for tumor growth than the constraint resulting from reduced access to arginine [266]. In addition, enforcing arginase activity in cancer cells might promote tumor progression by providing neoplastic cells with polyamines and other arginine subproducts that stimulate cell proliferation, and the tumor cells could adapt to limited arginine by upregulating amino acid transporters or by lowering their sensitivity to GCN2 and mTOR signaling. As described above, T cells have specific requirements for specific amino acids and arginine depletion activates GCN2 to block entry into S phase following T cell stimulation [232], suggesting that sufficient access to arginine is a prerequisite for cell cycle entry in T cells.

These mechanisms are also reminiscent of healing responses—used to control and prevent immune-mediated damage in inflamed and wounded tissues. A central hypothesis in current investigations is that malignant lesions hijack tissue healing pathways and subvert these mechanisms for suppressing anti-tumor immunity and creating potent pro-tumoral immune checkpoints. Macrophages and cells from the myeloid compartment are a paradigm of such hypotheses. M1 macrophages are regarded as mediators of resistance against tumor growth: they produce inflammatory cytokines, such as IL-1β and TNF-∝, which promote Th1 responses. M1 macrophages are also responsible for NO production [267], causing cell cycle arrest and tumor cell cytostasis or apoptosis, and further sensitizing cancer cells to TNF-induced cytotoxicity. On the contrary, macrophages conditioned by anti-inflammatory cytokines released in tumor microenvironment—such as IL-4, IL-10, amongst others—polarize towards the M2 phenotype [268]. M2-polarised macrophages induce Th2 responses and are characterized by the production of anti-inflammatory mediators, such as Arg1. Both in patients and mice, tumor-associated macrophages (TAMs) promote tumor progression and pro-tumoral tissue remodelling [267, 269]. Activated similar to M2 macrophages [105, 106], TAMs express substantial amounts of ARG1, leading to an over-production of ornithine, which can be used by ODC to synthesize polyamines, such as putrescine, spermidine, and spermine, and thus promote cancer cell proliferation and tumor vascularization [270, 271].

In parallel, murine and human neoplastic cells can secrete soluble factors—such as GM-CSF and G-CSF—that alter myelopoiesis and lead to the recruitment and accumulation of MDSCs [272]. For instance, increased numbers of circulating MDSCs exhibiting high Arg1 expression have been documented in glioblastoma patients [273]. In tumors, MDSCs frequently express Arg1 and reflect functional similarities to alternatively activated M2 macrophages [1, 274], including IL-10, TGF-β, and IDO expression [275]. Interestingly, it has been demonstrated that CD3ζ chain downregulation caused by tumor‐derived MDSCs overexpressing Arg1 might be more detrimental for CD4^+^ T cells than CD8^+^ T cells [276].

Counterintuitively, arginine-depleting immune cells might contribute to the establishment of barriers to natural and therapy-induced anti-tumor immunity, even if tumor cells do not catabolize arginine themselves. In light of these findings, arginine supplementation could seem counterproductive, feed-forwarding the establishment of an immune barrier. However, the combination of arginine supplementation with therapies that release other immunosuppressive barriers, such as PD-L1 blockade, has proven successful in preclinical models of osteosarcoma in mice [251]. Therefore, restoring arginine in the TME boosts anti-tumor T cells in a context, where the PD-1/PD-L1 axis is also disarmed for the in situ repression of T cells. Thus, and as discussed below, the exploitation of non-redundant immunosuppressive mechanisms bears great promise for the immediate future of cancer immunotherapies.

### Arginases as therapeutic solutions and targets in cancer

Numerous clinical trials have explored arginine depletion to combat different malignancies, such as lymphoblastic and acute myeloid leukemias, melanoma, as well as pancreatic and liver carcinomas [277]. These therapeutic strategies exploit a frequent ASS1 deficiency in tumor cells, which renders malignant cells auxotrophic for arginine [278]. Therefore, arginine deprivation in the bloodstream has been tested using the administration of two different pegylated proteins, the mycoplasma-derived arginine deiminase (ADI-Peg) or human Arginase 1 (Arg1-Peg).

Although ADI-Peg has demonstrated efficacious anti-tumor activity in ASS1-deficient tumors, such as HCC, ADI-Peg is significantly immunogenic and induces the production of blocking antibodies. Furthermore, ADI-Peg also leads to the synthesis of ammonia, a toxic product, causing neutropenia and neurological impairment [279]. Alternatively, Peg-Arg1 can also reduce arginine serum levels in vivo without inducing noticeable toxicity [280] and without evidence of immunogenicity. Consequently, Peg-Arg1 represents a safer and more efficacious therapy that has exhibited significant anti-tumor benefits in multiple preclinical cancer models and in cancer patients [277].

Arginase inhibitors have also been tested for cancer treatment. The most notorious case is that of the CB-1158 arginase inhibitor. In mouse preclinical models, CB-1158 released MDSC-mediated T cell immunosuppression and reduced tumor growth in a CD8^+^ T cell and NK cell-dependent manner [281]. Moreover, in preclinical models, CB-1158 also boosted the effect of standard-of-care immunotherapies—such PD-L1 checkpoint blockade, and adoptive T or NK cell therapies—and improved efficacy of the chemotherapeutic agent gemcitabine.

## Concluding remarks and perspectives

Early work in the arginine metabolism field focused on examining defects in arginine-starved immune cells. Subsequent studies identified key metabolic pathways driving arginine metabolism in immune cells and defined major roles of distinct immune cell lineages in immune responses to arginine depletion. Over the past decade, an increasingly refined picture has emerged of how cellular arginine metabolism affects the differentiation, expansion, and maturation of both macrophage, DC and T cell subsets. In parallel to a need for a better understanding of arginine metabolism in other immune cell lineages, some aspects of arginine relevance for immune cell biology remain unaddressed. Although intracellular arginine compartmentalization has been described, the mechanisms of arginine trafficking from the cytosol to key organelles, such as the mitochondrion, remain unknown. In addition, it is still unclear what mechanisms mediate arginine sensing in such organelles, a question of paramount importance considering the relevance of intracellular arginine metabolism for dictating immune outcomes.

It has also become evident that distinct arginine usages result in lineage- and subset-specific pro- or anti-inflammatory phenotypes. Translating and further expanding our knowledge on the metabolic fate of arginine is likely to lead to the development of new therapeutic strategies in many disease areas. Understanding how arginine metabolism integrates with other regulatory mechanisms, such as microRNA-155 upregulation in activated CD8 T cells, might reveal critical metabolic nodes that might be readily available for genetic or pharmacologic intervention. Given our growing appreciation of how arginine metabolism dictates divergent immune phenotypes, e.g., Arg2 limits T cell activation and effector functions but promotes T_reg_ metabolic fitness, integrating arginine metabolism and the inflammatory milieu in which T cells operate will be crucial for the development of next-generation immunotherapies. Isoform-specific arginase inhibitors or genetic engineering of arginine metabolic pathways could prove to be valuable therapeutic tools to favor immunogenic or tolerogenic responses in personalized immunotherapies.

## Abbreviations

AKT: Protein kinase B
AP-1: Activator protein 1
APRIL: A proliferation-inducing ligand
BH_4_: Tetrahydrobiopterin
C/EBPβ: CCAAT-enhancer-binding proteinβ
cAMP: Cyclic adenosine monophosphate
eIF2α: Eukaryotic translation initiation factor 2A
ERK: Extracellular signal-regulated kinase
FAD: Flavin adenine dinucleotide
FMN: Flavin mononucleotide
GM-CSF: Granulocyte–macrophage colony-stimulating factor
IFN: Interferon
IL: Interleukin
IL-2R: Interleukin 2 receptor
IRF1: Interferon regulatory factor 1
ITAM: Immunoreceptor tyrosine-based activation motif
L-NAME: l-n^g^-Nitroarginine methyl ester
L-NMMA: l-n^g^-Monomethyl arginine acetate
MHC: Major histocompatibility complex
NADPH: Nicotinamide adenine dinucleotide phosphate
NF-*κ*B: Nuclear factor kappa-light-chain-enhancer of activated B cells
pMHC-I: Cognate peptide and MHC-I molecule complex
PD-1: Programmed cell death protein 1
PGE2: Prostaglandin E_2_
PHA: Phytohemagglutinin
ROS: Reactive oxygen species
STAT: Signal transducer and activator of transcription
TGF-β: Transforming growth factor-beta
Th: T helper

## References

[1] Bronte V, Zanovello P (2005). Regulation of immune responses by l-arginine metabolism. Nat Rev Immunol.

[2] Nieves C, Langkamp-Henken B (2002). Arginine and immunity: a unique perspective. Biomed Pharmacother.

[3] Peranzoni E (2008). Role of arginine metabolism in immunity and immunopathology. Immunobiol.

[4] Murray PJ (2016). Amino acid auxotrophy as a system of immunological control nodes. Nat Immunol.

[5] Geiger R (2016). l-arginine modulates T cell metabolism and enhances survival and anti-tumor activity. Cell.

[6] Vincendeau P (2003). Arginases in parasitic diseases. Trends Parasitol.

[7] Yu YM (2001). Arginine and ornithine kinetics in severely burned patients: increased rate of arginine disposal. Am J Physiol-Endocrino Metab.

[8] Wilmore D (2004). Enteral and parenteral arginine supplementation to improve medical outcomes in hospitalized patients. J Nutr.

[9] Redmond HP (1998). Immunonutrition: the role of arginine.

[10] Scull CW, Rose WC (1930). Arginine metabolism I: the relation of the arginine content of the diet to the increments in tissue arginine during growth. J Biol Chem.

[11] Castillo L (1994). Plasma arginine kinetics in adult man: response to an arginine-free diet. Metabolism.

[12] Castillo L (1993). Plasma arginine and citrulline kinetics in adults given adequate and arginine-free diets. Proc Natl Acad Sci USA.

[13] Nakagawa I, Takahashi T, Suzuki T, Kobayashi K (1963). Amino acid requirements of children: minimal needs of tryptophan, arginine and histidine based on nitrogen balance method. J Nutr.

[14] Schulze E, Steiger E (1886). Ueber einen neuen stickstoffhaltigen Bestandtheil der Keimlinge von *Lupinus luteus*. Berichte der Dtsch Chem Gesellschaft.

[15] Hedin SG (1895). Eine Methode, das Lysin zu isoliren, nebst einigen Bemerkungen Über das Lysatinin. Z Physiol Chem.

[16] Krebs HA, Henseleit K (1932). Untersuchungen über die Harnstoffbildung im Tierkörper. Klin Wochenschr.

[17] Withers PC (1998). Urea: diverse functions of a ‘waste’ product. Clinical Experimental Pharmacol Physiol.

[18] Meijer AJ, Lamers WH, Chamuleau RAFM (1990). Nitrogen metabolism and ornithine cycle function. Physiol Rev.

[19] Newsholme P, Brennan L, Rubi B, Maechler P (2005). New insights into amino acid metabolism, β-cell function and diabetes. Clin Sci.

[20] Barbul A (1986). Arginine: biochemistry, physiology, and therapeutic implications. J Parenter Enter Nutr.

[21] Redmond HP, Daly JM (1993). Arginine. Nutr Immunol.

[22] Fitch CA, Platzer G, Okon M, Garcia-Moreno BE, McIntosh LP (2015). Arginine: its p*K*a value revisited. Protein Sci.

[23] Wallis M (1974). On the frequency of arginine in proteins and its implications for molecular evolution. Biochem Biophys Res Commun.

[24] King JL, Jukes TH (1969). Non-darwinian evolution. Sci.

[25] Barile MF, Leventhal BG (1968). Possible mechanism for mycoplasma inhibtion of lymphocyte transformation induced by phytohaemagglutinin. Nature.

[26] Osunkoya BO, Adler WH, Smith RT (1970). Effect of arginine deficiency on synthesis of DNA and immunoglobulin receptor of burkitt lymphoma cells. Nature.

[27] Barbul A, Rettura G, Levenson SM, Seifter E (1977). Arginine: a thymotropic and wound-healing promoting agent. Surg Forum.

[28] Tong BC, Barbul A (2004). Cellular and physiological effects of arginine. Mini Rev Med Chem.

[29] Mandal A (2006). Do malnutrition and nutritional supplementation have an effect on the wound healing process?. J Wound Care.

[30] Barbul A, Lazarou S, Efron D, Wasserkrug HL, Efron G (1990). Arginine enhances wound healing and lymphocyte immune response in humans. Surgery.

[31] Bansal V (2005). Interactions between fatty acids and arginine metabolism: implications for the design of immune-enhancing diets. J Parenter Enter Nutr.

[32] Grimble RF (2005). Immunonutrition. Curr Opin Gastroenterol.

[33] Ochoa JB, Makarenkova V, Bansal V (2004). A rational use of immune enhancing diets: when should we use dietary arginine supplementation?. Nutr Clinical Pract.

[34] Daly JM (1988). Immune and metabolic effects of arginine in the surgical patient. Ann Surg.

[35] Braga M (1996). Immune and nutritional effects of early enteral nutrition after major abdominal operations. Eur J Surg.

[36] Bower RH (1995). Early enteral administration of a formula (impactρ) supplemented with arginine, nucleotides, and fish oil in intensive care unit patients: results of a multicenter, prospective, randomized, clinical trial. Crit Care Med.

[37] Morris CR (2005). Dysregulated arginine metabolism, hemolysis-associated pulmonary hypertension, and mortality in sickle cell disease. JAMA.

[38] Powell-Tuck J (2007). Nutritional interventions in critical illness. Proc Nutr Soc.

[39] De Waele E, Malbrain MLNG, Spapen H (2020). Nutrition in sepsis: a bench-to-bedside review. Nutrients.

[40] Bode-Böger SM, Böger RH, Galland A, Tsikas D, Frölich JC (1998). l-arginine-induced vasodilation in healthy humans: pharmacokinetic-pharmacodynamic relationship. Br J Clin Pharmacol.

[41] Möller P, Alvestrand A, Bergström J, Fürst P, Hellström K (1983). Electrolytes and free amino acids in leg skeletal muscle of young and elderly women. Gerontology.

[42] Moller P, Bergstrom J, Eriksson S, Fürst P, Hellström K (1979). Effect of aging on free amino acids and electrolytes in leg skeletal muscle. Clin Sci.

[43] Wu G, Morris SM (1998). Arginine metabolism: nitric oxide and beyond. Biochem J.

[44] Visek WJ (1986). Arginine needs, physiological state and usual diets. Reevaluation J Nutr.

[45] Mirmiran P, Moghadam SK, Bahadoran Z, Ghasemi A, Azizi F (2017). Dietary l-arginine intakes and the risk of metabolic syndrome: a 6-year follow-up in tehran lipid and glucose study. Prev Nutr Food Sci.

[46] White MF (1985). The transport of cationic amino acids across the plasma membrane of mammalian cells. BBA—Rev Biomembr.

[47] Mann GE, Yudilevich DL, Sobrevia L (2003). Regulation of amino acid and glucose transporters in endothelial and smooth muscle cells. Physiol Rev.

[48] Castillo L (1993). Dietary arginine uptake by the splanchnic region in adult humans. Am J Physiol Endocrinol Metab.

[49] Barbul A, Uliyargoli A (2007). Use of exogenous arginine in multiple organ dysfunction syndrome and sepsis. Crit Care Med.

[50] Cheung CW, Cohen NS, Raijman L (1989). Channeling of urea cycle intermediates in situ in permeabilized hepatocytes. J Biol Chem.

[51] Watford M (1991). The urea cycle: a two-compartment system. Essays Biochem.

[52] Castillo L, Beaumier L, Ajami AM, Young VR (1996). Whole body nitric oxide synthesis in healthy men determined from [^15^N]arginine-to-[^15^N]citrulline labeling. Proc Natl Acad Sci USA.

[53] Rose WC, Haines WJ, Warner DT (1954). The amino acid requirements of man. V. The role of lysine, arginine, and tryptophan. J Biol Chem.

[54] Featherston WR, Rogers QR, Freedland RA (1973). Relative importance of kidney and liver in synthesis of arginine by the rat. Am J Physiol.

[55] Dhanakoti SN, Brosnan JT, Herzberg GR, Brosnan ME (1990). Renal arginine synthesis: studies in vitro and in vivo. Am J Physiol-Endocrinol Metab.

[56] Dhanakoti SN, Brosnan ME, Herzberg GR, Brosnan JT (1992). Cellular and subcellular localization of enzymes of arginine metabolism in rat kidney. Biochem J.

[57] Windmueller HG, Spaeth AE (1981). Source and fate of circulating citrulline. Am J Physiol-Endocrinol Metab.

[58] Rabier D, Kamoun P (1995). Metabolism of citrulline in man. Amino Acids.

[59] Wu G, Brosnan JT (1992). Macrophages can convert citrulline into arginine. Biochem J.

[60] Nussler AK, Billiar TR, Liu ZZ, Morris SM (1994). Coinduction of nitric oxide synthase and argininosuccinate synthetase in a murine macrophage cell line implications for regulation of nitric oxide production. J Biol Chem.

[61] Baydoun AR, Emery PW, Pearson JD, Mann GE (1990). Substrate-dependent regulation of intracellular amino acid concentrations in cultured bovine aortic endothelial cells. Biochem Biophys Res Commun.

[62] Bogle RG, MacAllister RJ, Whitley GSJ, Vallance P (1995). Induction of *N*(G)-monomethyl-l-arginine uptake: a mechanism for differential inhibition of NO synthases?. Am J Physiol—Cell Physiol.

[63] Böger RH (2000). LDL cholesterol upregulates synthesis of asymmetrical dimethylarginine in human endothelial cells: involvement of *S*-adenosylmethionine-dependent methyltransferases. Circ Res.

[64] Topal G, Brunet A, Walch L, Boucher J-L, David-Dufilho M (2006). Mitochondrial arginase II modulates nitric-oxide synthesis through nonfreely exchangeable l-arginine pools in human endothelial cells. J Pharmacol Exp Ther.

[65] Palacín M, Estévez R, Bertran J, Zorzano A (1998). Molecular biology of mammalian plasma membrane amino acid transporters. Physiol Rev.

[66] Closs EI (1996). CATs, a family of three distinct mammalian cationic amino acid transporters. Amino Acids.

[67] Devés R, Boyd CAR (1998). Transporters for cationic amino acids in animal cells: discovery, structure, and function. Physiol Rev.

[68] Closs EI, Boissel JP, Habermeier A, Rotmann A (2006). Structure and function of cationic amino acid transporters (CATs). J Membr Biol.

[69] Closs EI, Gräf P, Habermeier A, Cunningham JM, Förstermann U (1997). Human cationic amino acid transporters hCAT-1, hCAT-2A, and hCAT-2B: three related carriers with distinct transport properties. Biochem.

[70] Jungnickel KEJ, Parker JL, Newstead S (2018). Structural basis for amino acid transport by the CAT family of SLC7 transporters. Nat Commun.

[71] Bogle RG, Moncada S, Pearson JD, Mann GE (1992). Identification of inhibitors of nitric oxide synthase that do not interact with the endothelial cell l-arginine transporter. Br J Pharmacol.

[72] Schmidt K, Klatt P, Mayer B (1993). Characterization of endothelial cell amino acid transport systems involved in the actions of nitric oxide synthase inhibitors. Mol Pharmacol.

[73] DeGeorge GL, Heck DE, Laskin JD (1997). Arginine metabolism in keratinocytes and macrophages during nitric oxide biosynthesis. Biochem Pharmacol.

[74] Rodriguez PC (2004). Arginase I production in the tumor microenvironment by mature myeloid cells inhibits T-cell receptor expression and antigen-specific T-cell responses. Cancer Res.

[75] MacLeod CL, Kakuda DK (1996). Regulation of CAT: cationic amino acid transporter gene expression. Amino Acids.

[76] Kakuda DK, Finley KD, Maruyama M, MacLeod CL (1998). Stress differentially induces cationic amino acid transporter gene expression. Biochim Biophys Acta-Biomembr.

[77] Cendan JC, Souba WW, Copeland EM, Lind DS (1995). Characterization and growth factor stimulation of l-arginine transport in a human colon cancer cell line. Ann Surg Oncol.

[78] Cendan JC (1996). Inflammatory mediators stimulate arginine transport and arginine-derived nitric oxide production in a murine breast cancer cell line. J Surg Res.

[79] Porcelli V, Fiermonte G, Longo A, Palmieri F (2014). The human gene SLC25A29, of solute carrier family 25, encodes a mitochondrial transporter of basic amino acids. J Biol Chem.

[80] Kim HS, Cha SY, Jo CH, Han A, Hwang KY (2014). The crystal structure of arginyl-tRNA synthetase from *Homo sapiens*. FEBS Lett.

[81] Ferber S, Ciechanover A (1987). Role of arginine-tRNA in protein degradation by the ubiquitin pathway. Nature.

[82] Vockley JG (1996). Cloning and characterization of the human type II arginase gene. Genomics.

[83] Gotoh T (1996). Molecular cloning of cDNA for nonhepatic mitochondrial arginase (arginase II) and comparison of its induction with nitric oxide synthase in a murine macrophage-like cell line. FEBS Lett.

[84] Jenkinson CP, Grody WW, Cederbaum SD (1996). Comparative properties of arginases. Comp Biochem Physiol B.

[85] Morris SM (2010). Arginine: master and commander in innate immune responses. Sci Signal.

[86] Nong Z, Hoylaerts M, Van Pelt N, Collen D, Janssens S (1997). Nitric oxide inhalation inhibits platelet aggregation and platelet-mediated pulmonary thrombosis in rats. Circ Res.

[87] Hickey MJ, Kubes P (1999). Nitric oxide and leukocyte adhesion: experience with NO inhibitors, NO donors and iNOS-deficient mice. Shock, sepsis, and organ failure.

[88] Baek KJ, Thiel BA, Lucas S, Stuehr DJ (1993). Macrophage nitric oxide synthase subunits. Purification, characterization, and role of prosthetic groups and substrate in regulating their association into a dimeric enzyme. J Biol Chem.

[89] Vos TA (1997). Differential effects of nitric oxide synthase inhibitors on endotoxin-induced liver damage in rats. Gastroenterol.

[90] Kimura H (1998). Increased expression of an inducible isoform of nitric oxide synthase and the formation of peroxynitrite in colonic mucosa of patients with active ulcerative colitis. Gut.

[91] Forrester K (1996). Nitric oxide-induced p53 accumulation and regulation of inducible nitric oxide synthase expression by wild-type p53. Proc Natl Acad Sci USA.

[92] Murphy MP (1999). Nitric oxide and cell death. Biochim Biophys Acta.

[93] Sarti P, Forte E, Mastronicola D, Giuffrè A, Arese M (2012). Cytochrome c oxidase and nitric oxide in action: molecular mechanisms and pathophysiological implications. Biochim Biophys Acta.

[94] Griffith OW, Stuehr DJ (1995). Nitric oxide synthases: properties and catalytic mechanism. Annu Rev Physiol.

[95] Lee J, Ryu H, Ferrante RJ, Morris SM, Ratan RR (2003). Translational control of inducible nitric oxide synthase expression by arginine can explain the arginine paradox. Proc Natl Acad Sci USA.

[96] Fligger J, Blum J, Jungi TW (1999). Induction of intracellular arginase activity does not diminish the capacity of macrophages to produce nitric oxide in vitro. Immunobiology.

[97] Förstermann U (1994). Nitric oxide synthase isozymes. Characterization, purification, molecular cloning, and functions. Hypertension.

[98] Daghigh F, Fukuto JM, Ash DE (1994). Inhibition of rat liver arginase by an intermediate in NO biosynthesis, NG-hydroxy-l-arginine: implications for the regulation of nitric oxide biosynthesis by arginase. Biochem Biophys Res Commun.

[99] Wyss M, Kaddurah-Daouk R (2000). Creatine and creatinine metabolism. Physiol Rev.

[100] Morris SM (2007). Arginine metabolism: boundaries of our knowledge. J Nutr.

[101] Li G (1994). Agmatine: an endogenous clonidine—displacing substance in the brain. Science.

[102] Morrissey J, Mccracken R, Ishidoya S, Klahr S (1995). Partial cloning and characterization of an arginine decarboxylase in the kidney. Kidney Int.

[103] Lortie MJ (1996). Agmatine, a bioactive metabolite of arginine: production, degradation, and functional effects in the kidney of the rat. J Clin Invest.

[104] Sastre M, Galea E, Feinstein D, Reis DJ, Regunathan S (1998). Metabolism of agmatine in macrophages: modulation by lipopolysaccharide and inhibitory cytokines. Biochem J.

[105] Noël W, Raes G, Ghassabeh GH, De Baetselier P, Beschin A (2004). Alternatively activated macrophages during parasite infections. Trends Parasitol.

[106] Gordon S (2003). Alternative activation of macrophages. Nat Rev Immunol.

[107] Gallina G (2006). Tumors induce a subset of inflammatory monocytes with immunosuppressive activity on CD8^+^ T cells. J Clin Invest.

[108] Lumeng CN, Bodzin JL, Saltiel AR (2007). Obesity induces a phenotypic switch in adipose tissue macrophage polarization. J Clin Invest.

[109] Thomas AC, Mattila JT (2014). ‘Of mice and men’: arginine metabolism in macrophages. Frontiers Immunol.

[110] Bronte V, Serafini P, Mazzoni A, Segal DM, Zanovello P (2003). l-Arginine metabolism in myeloid cells controls T-lymphocyte functions. Trends Immunol.

[111] Louis CA (1998). Distinct arginase isoforms expressed in primary and transformed macrophages: regulation by oxygen tension. Am J Physiol.

[112] Buga GM (1996). Arginase activity in endothelial cells: inhibition by *N*(G)-hydroxy-l- arginine during high-output NO production. Am J Physiol.

[113] Louis CA, Mody V, Henry WL, Reichner JS, Albina JE (1999). Regulation of arginase isoforms I and II by IL-4 in cultured murine peritoneal macrophages. Am J Physiol.

[114] Fernández-Ruiz V, López-Moratalla N, González A (2005). Production of nitric oxide and self-nitration of proteins during monocyte differentiation to dendritic cells. J Physiol Biochem.

[115] Ji L (2019). Slc6a8-mediated creatine uptake and accumulation reprogram macrophage polarization via regulating cytokine responses article Slc6a8-mediated creatine uptake and accumulation reprogram macrophage polarization via regulating cytokine responses. Immunity.

[116] Kim JH, Kim JY, Mun CH, Suh M, Lee JE (2017). Agmatine modulates the phenotype of macrophage acute phase after spinal cord injury in rats. Exp Neurobiol.

[117] Regunathan S, Piletz JE (2003). Regulation of inducible nitric oxide synthase and agmatine synthesis in macrophages and astrocytes. Ann New York Academy Sci.

[118] Chai J (2016). Agmatine reduces lipopolysaccharide-mediated oxidant response via activating PI3K/Akt pathway and up-regulating Nrf2 and HO-1 expression in macrophages. PLoS ONE.

[119] Arias M (1997). Inhibition of virulent *Mycobacterium tuberculosis* by Bcg(r) and Bcg(s) macrophages correlates with nitric oxide production. J Infect Dis.

[120] Gross A (1998). Expression and bactericidal activity of nitric oxide synthase in *Brucella suis*-infected murine macrophages. Infect Immun.

[121] Silva JS, Vespa GNR, Cardoso MAG, Aliberti JCS, Cunha FQ (1995). Tumor necrosis factor alpha mediates resistance to *Trypanosoma cruzi* infection in mice by inducing nitric oxide production in infected gamma interferon-activated macrophages. Infect Immun.

[122] Miyagi K, Kawakami K, Saito A (1997). Role of reactive nitrogen and oxygen intermediates in gamma interferon—stimulated murine macrophage bactericidal activity against *Burkholderia pseudomallei*. Infect Immun.

[123] Kleinert H (1998). Cytokine induction of NO synthase II in human DLD-1 cells: roles of the JAK-STAT, AP-1 and NF-κB-signaling pathways. Br J Pharmacol.

[124] Ganster RW, Taylor BS, Shao L, Geller DA (2001). Complex regulation of human inducible nitric oxide synthase gene transcription by Stat 1 and NF-κB. Proc Natl Acad Sci USA.

[125] Modolell M, Corraliza IM, Link F, Soler G, Eichmann K (1995). Reciprocal regulation of the nitric oxide synthase/arginase balance in mouse bone marrow-derived macrophages by TH 1 and TH 2 cytokines. Eur J Immunol.

[126] Martin E, Nathan C, Xie QW (1994). Role of interferon regulatory factor 1 in induction of nitric oxide synthase. J Exp Med.

[127] Wiesinger H (2001). Arginine metabolism and the synthesis of nitric oxide in the nervous system. Prog Neurobiol.

[128] Kakuda DK, Sweet MJ, MacLeod CL, Hume DA, Markovich D (1999). CAT2-mediated l-arginine transport and nitric oxide production in activated macrophages. Biochem J.

[129] Sakai N, Kaufman S, Milstien S (1995). Parallel induction of nitric oxide and tetrahydrobiopterin synthesis by cytokines in rat glial cells. J Neurochem.

[130] Nussler AK (1996). A cohort of supporting metabolic enzymes is coinduced with nitric oxide synthase in human tumor cell lines. Cancer Lett.

[131] Frank S, Madlener M, Pfeilschifter J, Werner S (1998). Induction of inducible nitric oxide synthase and its corresponding tetrahydrobiopterin-cofactor-synthesizing enzyme GTP-cyclohydrolase I during cutaneous wound repair. J Invest Dermatol.

[132] García-Nogales P, Almeida A, Fernández E, Medina JM, Bolaños JP (1999). Induction of glucose-6-phosphate dehydrogenase by lipopolysaccharide contributes to preventing nitric oxide-mediated glutathione depletion in cultured rat astrocytes. J Neurochem.

[133] Qualls JE (2012). Sustained generation of nitric oxide and control of mycobacterial infection requires argininosuccinate synthase 1. Cell Host Microbe.

[134] Vasquez-Dunddel D (2013). STAT3 regulates arginase-i in myeloid-derived suppressor cells from cancer patients. J Clin Invest.

[135] Gray MJ, Poljakovic M, Kepka-Lenhart D, Morris SM (2005). Induction of arginase I transcription by IL-4 requires a composite DNA response element for STAT6 and C/EBPβ. Gene.

[136] Pauleau A-L (2004). Enhancer-mediated control of macrophage-specific arginase I expression. J Immunol.

[137] Qualls JE (2010). Arginine usage in mycobacteria-infected macrophages depends on autocrine-paracrine cytokine signaling. Sci Signal.

[138] Munder M (1999). Th1/Th2-regulated expression of arginase isoforms in murine macrophages and dendritic cells. J Immunol.

[139] Rodriguez PC (2005). Arginase I in myeloid suppressor cells is induced by COX-2 in lung carcinoma. J Exp Med.

[140] Bernard AC (2000). Beta adrenoceptor regulation of macrophage arginase activity. Surgery.

[141] Zheng S, McElwain CM, Taffet SM (1991). Regulation of mouse ornithine decarboxylase gene expression in a macrophage-like cell line: synergistic induction by bacterial lipopolysaccharide and camp. Biochem Biophys Res Commun.

[142] Morris SM, Kepka-Lenhart D, Chen LIC (1998). Differential regulation of arginases and inducible nitric oxide synthase in murine macrophage cells. Am J Physiol.

[143] Barron L (2013). Role of arginase 1 from myeloid cells in Th2-dominated lung inflammation. PLoS ONE.

[144] Campbell L, Saville CR, Murray PJ, Cruickshank SM, Hardman MJ (2013). Local arginase 1 activity is required for cutaneous wound healing. J Invest Dermatol.

[145] Shearer JD, Richards JR, Mills CD, Caldwell MD (1997). Differential regulation of macrophage arginine metabolism: a proposed role in wound healing. Am J Physiol.

[146] Albina JE, Abate JA, Mastrofrancesco B (1993). Role of ornithine as a proline precursor in healing wounds. J Surg Res.

[147] Albina JE, Mills CD, Henry WL, Caldwell MD (1990). Temporal expression of different pathways of l-arginine metabolism in healing wounds. J Immunol.

[148] Esser-von Bieren J (2013). Antibodies trap tissue migrating helminth larvae and prevent tissue damage by driving IL-4Rα-independent alternative differentiation of macrophages. PLoS Pathog.

[149] Bussière FI (2005). Spermine causes loss of innate immune response to *Helicobacter pylori* by inhibition of inducible nitric-oxide synthase translation. J Biol Chem.

[150] Mössner J, Hammermann R, Racké K (2001). Concomitant down-regulation of l-arginine transport and nitric oxide (NO) synthesis in rat alveolar macrophages by the polyamine spermine. Pulm Pharmacol Ther.

[151] Pesce JT (2009). Arginase-1-expressing macrophages suppress Th2 cytokine-driven inflammation and fibrosis. PLoS Pathog.

[152] Herbert DR (2010). Arginase I suppresses IL-12/IL-23p40-driven intestinal inflammation during acute schistosomiasis. J Immunol.

[153] Mattila JT (2013). Microenvironments in tuberculous granulomas are delineated by distinct populations of macrophage subsets and expression of nitric oxide synthase and arginase isoforms. J Immunol.

[154] Monin L (2015). Helminth-induced arginase-1 exacerbates lung inflammation and disease severity in tuberculosis. J Clin Invest.

[155] El Kasmi KC (2008). Toll-like receptor-induced arginase 1 in macrophages thwarts effective immunity against intracellular pathogens. Nat Immunol.

[156] Gobert AP (2001). *Helicobacter pylori* arginase inhibits nitric oxide production by eukaryotic cells: a strategy for bacterial survival. Proc Natl Acad Sci.

[157] Kapp K (2014). Granulocyte functions are independent of arginine availability. J Leukoc Biol.

[158] Munder M (2006). Suppression of T-cell functions by human granulocyte arginase. Blood.

[159] Jacobsen LC, Theilgaard-Mönch K, Christensen EI, Borregaard N (2007). Arginase 1 is expressed in myelocytes/metamyelocytes and localized in gelatinase granules of human neutrophils. Blood.

[160] Rotondo R (2011). Exocytosis of azurophil and arginase 1-containing granules by activated polymorphonuclear neutrophils is required to inhibit T lymphocyte proliferation. J Leukoc Biol.

[161] Darcy CJ (2014). Neutrophils with myeloid derived suppressor function deplete arginine and constrain T cell function in septic shock patients. Crit Care.

[162] Sakiniene E, Bremell T, Tarkowski A (1997). Inhibition of nitric oxide synthase (NOS) aggravates *Staphylococcus aureus* septicaemia and septic arthritis. Clin Exp Immunol.

[163] Nagarkoti S (2019). l-Arginine and tetrahydrobiopterin supported nitric oxide production is crucial for the microbicidal activity of neutrophils. Free Radic Res.

[164] Ródenas J, Mitjavila MT, Carbonell T (1998). Nitric oxide inhibits superoxide production by inflammatory polymorphonuclear leukocytes. Am J Physiol.

[165] Nath J, Powledge A (1997). Modulation of human neutrophil inflammatory responses by nitric oxide: studies in unprimed and LPS-primed cells. J Leukoc Biol.

[166] Ostrand-Rosenberg S, Fenselau C (2018). Myeloid-derived suppressor cells: immune-suppressive cells that impair antitumor immunity and are sculpted by their environment. J Immunol.

[167] Serafini P, Borrello I, Bronte V (2006). Myeloid suppressor cells in cancer: recruitment, phenotype, properties, and mechanisms of immune suppression. Semin Cancer Biol.

[168] Brys L (2005). Reactive oxygen species and 12/15-lipoxygenase contribute to the antiproliferative capacity of alternatively activated myeloid cells elicited during helminth infection. J Immunol.

[169] Mazzoni A (2002). Myeloid suppressor lines inhibit T cell responses by an NO-dependent mechanism. J Immunol.

[170] Tsuei BJ (2001). Surgery induces human mononuclear cell arginase 1 expression. J Trauma.

[171] Ochoa JB (2001). Arginase I expression and activity in human mononuclear cells after injury. Ann Surg.

[172] Makarenkova VP, Bansal V, Matta BM, Perez LA, Ochoa JB (2006). CD11b + /Gr-1 + myeloid suppressor cells cause T cell dysfunction after traumatic stress. J Immunol.

[173] Bronte V (2003). IL-4-induced arginase 1 suppresses alloreactive T cells in tumor-bearing mice. J Immunol.

[174] Zea AH (2005). Arginase-producing myeloid suppressor cells in renal cell carcinoma patients: a mechanism of tumor evasion. Cancer Res.

[175] Bronte V (2005). Boosting antitumor responses of T lymphocytes infiltrating human prostate cancers. J Exp Med.

[176] Kusmartsev S, Gabrilovich DI (2006). Role of immature myeloid cells in mechanisms of immune evasion in cancer. Cancer Immunol Immunother.

[177] Rutschman R (2001). Cutting edge: Stat6-dependent substrate depletion regulates nitric oxide production. J Immunol.

[178] Serafini P, Mgebroff S, Noonan K, Borrello I (2008). Myeloid-derived suppressor cells promote cross-tolerance in B-cell lymphoma by expanding regulatory T cells. Cancer Res.

[179] Rodriguez PC (2003). l-Arginine consumption by macrophages modulates the expression of CD3 chain in T lymphocytes. J Immunol.

[180] Verrey F (2004). CATs and HATs: The SLC7 family of amino acid transporters. Pflugers Arch.

[181] Kaneko S (2007). Ornithine transport via cationic amino acid transporter-1 is involved in ornithine cytotoxicity in retinal pigment epithelial cells. Investig Ophthalmol Vis Sci.

[182] White AR (2006). Knockdown of arginase I restores NO signaling in the vasculature of old rats. Hypertension.

[183] Xia Y, Zweier JL (1997). Superoxide and peroxynitrite generation from inducible nitric oxide synthase in macrophages. Proc Natl Acad Sci USA.

[184] Xia Y, Roman LJ, Masters BSS, Zweier JL (1998). Inducible nitric-oxide synthase generates superoxide from the reductase domain. J Biol Chem.

[185] Takahashi A (2005). Preferential cell death of CD8^+^ effector memory (CCR7 − CD45RA − ) T cells by hydrogen peroxide-induced oxidative stress. J Immunol.

[186] Case AJ (2011). Elevated mitochondrial superoxide disrupts normal T cell development, impairing adaptive immune responses to an influenza challenge. Free Radic Biol Med.

[187] Kesarwani P, Murali AK, Al-Khami AA, Mehrotra S (2013). Redox regulation of T-cell function: from molecular mechanisms to significance in human health and disease. Antioxid Redox Signal.

[188] Nagaraj S (2007). Altered recognition of antigen is a mechanism of CD8^+^ T cell tolerance in cancer. Nat Med.

[189] Molon B (2011). Chemokine nitration prevents intratumoral infiltration of antigen-specific T cells. J Exp Med.

[190] Lee DH (2016). Glutathione peroxidase 1 deficiency attenuates concanavalin A-induced hepatic injury by modulation of T-cell activation. Cell Death Dis.

[191] Baniyash M (2004). TCR ζ-chain downregulation: curtailing an excessive inflammatory immune response. Nat Rev Immunol.

[192] Macagno A, Napolitani G, Lanzavecchia A, Sallusto F (2007). Duration, combination and timing: the signal integration model of dendritic cell activation. Trends Immunol.

[193] Banchereau J, Steinman RM (1998). Dendritic cells and the control of immunity. Nature.

[194] Dunand-Sauthier I (2011). Silencing of c-Fos expression by microRNA-155 is critical for dendritic cell maturation and function. Blood.

[195] Liscovsky MV (2009). Interferon-γ priming is involved in the activation of arginase by oligodeoxinucleotides containing CpG motifs in murine macrophages. Immunology.

[196] Dunand-Sauthier I (2014). Repression of arginase-2 expression in dendritic cells by microRNA-155 is critical for promoting T cell proliferation. J Immunol.

[197] Geissmann F (2008). Blood monocytes: distinct subsets, how they relate to dendritic cells, and their possible roles in the regulation of T-cell responses. Immunol Cell Biol.

[198] Serbina NV, Salazar-Mather TP, Biron CA, Kuziel WA, Pamer EG (2003). TNF/iNOS-producing dendritic cells mediate innate immune defense against bacterial infection. Immunity.

[199] Copin R, De Baetselier P, Carlier Y, Letesson J-J, Muraille E (2007). MyD88-dependent activation of B220−CD11b+LY-6C+ dendritic cells during *Brucella melitensis* infection. J Immunol.

[200] De Trez C (2009). iNOS-producing inflammatory dendritic cells constitute the major infected cell type during the chronic *Leishmania* major infection phase of C57BL/6 resistant mice. PLoS Pathog.

[201] Marigo I (2016). T cell cancer therapy requires CD40-CD40L activation of tumor necrosis factor and inducible nitric-oxide-synthase-producing dendritic cells. Cancer Cell.

[202] Kania G (2013). Innate signaling promotes formation of regulatory nitric oxide-producing dendritic cells limiting T-cell expansion in experimental autoimmune myocarditis. Circulation.

[203] Guilliams M (2009). IL-10 dampens TNF/inducible nitric oxide synthase-producing dendritic cell-mediated pathogenicity during parasitic infection. J Immunol.

[204] Mondanelli G (2017). A relay pathway between arginine and tryptophan metabolism confers immunosuppressive properties on dendritic cells. Immunity.

[205] McGovern N (2017). Human fetal dendritic cells promote prenatal T-cell immune suppression through arginase-2. Nature.

[206] Yang J, Gonon AT, Sjöquist PO, Lundberg JO, Pernow J (2013). Arginase regulates red blood cell nitric oxide synthase and export of cardioprotective nitric oxide bioactivity. Proc Natl Acad Sci USA.

[207] Kim PS (2002). Expression of the liver form of arginase in erythrocytes. Mol Genet Metab.

[208] Delyea C (2018). CD71+ erythroid suppressor cells promote fetomaternal tolerance through arginase-2 and PDL-1. J Immunol.

[209] Elahi S (2013). Immunosuppressive CD71+ erythroid cells compromise neonatal host defence against infection. Nature.

[210] Dunsmore G (2017). Erythroid suppressor cells compromise neonatal immune response against bordetella pertussis. J Immunol.

[211] Namdar A (2017). CD71+ erythroid suppressor cells impair adaptive immunity against bordetella pertussis. Sci Rep.

[212] Hsueh EC (2012). Deprivation of arginine by recombinant human arginase in prostate cancer cells. J Hematol Oncol.

[213] Peters H, Border WA, Noble NA (1999). l-Arginine supplementation increases mesangial cell injury and subsequent tissue fibrosis in experimental glomerulonephritis. Kidney Int.

[214] Popovic PJ, Ochoa JB (2007). Arginine and immunity 1–3. J Nutr.

[215] Martí i Líndez A-AA (2019). Mitochondrial arginase-2 is a cell-autonomous regulator of CD8+ T cell function and antitumor efficacy. JCI Insight.

[216] Ochoa JB (2001). Effects of l-arginine on the proliferation of T lymphocyte subpopulations. J Parenter Enter Nutr.

[217] Barbul A (1985). Intravenous hyperalimentation with high arginine levels improves wound healing and immune function. J Surg Res.

[218] Calder PC, Yaqoob P 2003 Amino acids and immune function. Metabolic and Therapeutic Aspects of Amino Acids in Clinical Nutrition, 2nd Edn

[219] Weissman AM (1988). Tyrosine phosphorylation of the human T cell antigen receptor zeta-chain: activation via CD3 but not CD2. J Immunol..

[220] Rodriguez PC (2002). Regulation of T cell receptor CD3zeta chain expression by l-arginine. J Biol Chem.

[221] Minami Y, Weissman AM, Samelson LE, Klausner RD (1987). Building a multichain receptor: synthesis, degradation, an assembly of the T-cell antigen receptor. Proc Natl Acad Sci USA.

[222] Kwon H, Spencer TE, Bazer FW, Wu G (2003). Developmental changes of amino acids in ovine fetal fluids. Biol Reprod.

[223] Bansal V (2004). Citrulline can preserve proliferation and prevent the loss of CD3ζ chain under conditions of low arginine. J Parenter Enter Nutr.

[224] Grzywa TM (2020). Myeloid cell-derived arginase in cancer immune response. Front Immunol.

[225] Zabaleta J (2004). Helicobacter pylori arginase inhibits T cell proliferation and reduces the expression of the TCR zeta-chain (CD3zeta). J Immunol.

[226] Feldmeyer N (2012). Arginine deficiency leads to impaired cofilin dephosphorylation in activated human T lymphocytes. Int Immunol.

[227] Ghosh M (2004). Cofilin promotes actin polymerization and defines the direction of cell motility. Science.

[228] Huang Y, Burkhardt JK (2007). T-cell-receptor-dependent actin regulatory mechanisms. J Cell Sci.

[229] Zea AH (2004). l-Arginine modulates CD3ζ expression and T cell function in activated human T lymphocytes. Cell Immunol.

[230] Kato JY (1997). Control of G1 progression by D-type cyclins: key event for cell proliferation. Leukemia.

[231] Rodriguez PC (2010). l-Arginine deprivation regulates cyclin D3 mRNA stability in human T cells by controlling HuR expression. J Immunol.

[232] Rodriguez PC, Quiceno DG, Ochoa AC (2007). l-Arginine availability regulates T-lymphocyte cell-cycle progression. Blood.

[233] Holcik M, Sonenberg N (2005). Translational control in stress and apoptosis. Nat Rev Mol Cell Biol.

[234] Fallarino F (2006). The combined effects of tryptophan starvation and tryptophan catabolites down-regulate T cell receptor *ζ*-chain and induce a regulatory phenotype in naive T cells. J Immunol.

[235] Munn DH (2005). GCN2 kinase in T cells mediates proliferative arrest and anergy induction in response to indoleamine 2,3-dioxygenase. Immunity.

[236] Morrow K (2013). Anti-leukemic mechanisms of pegylated arginase I in acute lymphoblastic T-cell leukemia. Leukemia.

[237] Fletcher M (2015). l-Arginine depletion blunts antitumor T-cell responses by inducing myeloid-derived suppressor cells. Cancer Res.

[238] Fultang L (2020). Metabolic engineering against the arginine microenvironment enhances CAR-T cell proliferation and therapeutic activity. Blood.

[239] Lowe MM (2019). Regulatory T cells use arginase 2 to enhance their metabolic fitness in tissues. JCI Insight.

[240] Saio M, Radoja S, Marino M, Frey AB (2001). Tumor-infiltrating macrophages induce apoptosis in activated CD8+ T cells by a mechanism requiring cell contact and mediated by both the cell-associated form of TNF and nitric oxide. J Immunol.

[241] Hildeman DA (1999). Reactive oxygen species regulate activation-induced T cell apoptosis. Immunity.

[242] Hildeman DA (2003). Control of Bcl-2 expression by reactive oxygen species. Proc Natl Acad Sci USA.

[243] Vig M (2004). Inducible nitric oxide synthase in T cells regulates T cell death and immune memory. J Clin Invest.

[244] Fischer TA (2001). Activation of cGMP-dependent protein kinase Iβ inhibits interleukin 2 release and proliferation of T cell receptor-stimulated human peripheral T cells. J Biol Chem.

[245] Macphail SE (2003). Nitric oxide regulation of human peripheral blood mononuclear cells: critical time dependence and selectivity for cytokine versus chemokine expression. J Immunol.

[246] Bingisser RM, Tilbrook PA, Holt PG, Kees UR (1998). Macrophage-derived nitric oxide regulates T cell activation via reversible disruption of the Jak3/STAT5 signaling pathway. J Immunol..

[247] Miyazaki T (1994). Functional activation of Jak1 and Jak3 by selective association with IL-2 receptor subunits. Science.

[248] Lu T (2011). Tumor-infiltrating myeloid cells induce tumor cell resistance to cytotoxic T cells in mice. J Clin Invest.

[249] Kazak L, Cohen P (2020). Creatine metabolism: energy homeostasis, immunity and cancer biology. Nat Rev Endocrinol.

[250] Di Biase S (2019). Creatine uptake regulates CD8 T cell antitumor immunity. J Exp Med.

[251] He X, Lin H, Yuan L, Li B (2017). Combination therapy with l-arginine and α-PD-L1 antibody boosts immune response against osteosarcoma in immunocompetent mice. Cancer Biol Ther.

[252] De Jonge WJ (2002). Arginine deficiency affects early B cell maturation and lymphoid organ development in transgenic mice. J Clin Invest.

[253] Kobayashi T (1998). Arginine enhances induction of *t* helper 1 and *t* helper 2 cytokine synthesis by peyer’s patch αβ T cells and antigen-specific mucosal immune response. Biosci Biotechnol Biochem.

[254] Miret JJ (2019). Suppression of myeloid cell arginase activity leads to therapeutic response in a NSCLC mouse model by activating anti-tumor immunity. J Immunother Cancer.

[255] Saini AS, Shenoy GN, Rath S, Bal V, George A (2014). Inducible nitric oxide synthase is a major intermediate in signaling pathways for the survival of plasma cells. Nat Immunol.

[256] Park KGM (1991). Stimulation of lymphocyte natural cytotoxicity by l-arginine. Lancet.

[257] Brittenden J (1994). l-Arginine stimulates host defenses in patients with breast cancer. Surgery.

[258] Reynolds JV (1988). Immunomodulatory mechanisms of arginine. Surgery.

[259] Lamas B (2012). Altered functions of natural killer cells in response to l-arginine availability. Cell Immunol.

[260] Oberlies J (2009). Regulation of NK cell function by human granulocyte arginase. J Immunol.

[261] Goh CC (2016). Hepatitis C virus-induced myeloid-derived suppressor cells suppress NK cell IFN-γ production by altering cellular metabolism via arginase-1. J Immunol.

[262] Furuke K (1999). Human NK cells express endothelial nitric oxide synthase, and nitric oxide protects them from activation-induced cell death by regulating expression of TNF-α. J Immunol.

[263] Filep JG, Baron C, Lachance S, Perreault C, Chan JSD (1996). Involvement of nitric oxide in target-cell lysis and DNA fragmentation induced by murine natural killer cells. Blood.

[264] Cifone MG (1994). Induction of the nitric oxide-synthesizing pathway in fresh and interleukin 2-cultured rat natural killer cells. Cell Immunol.

[265] Tate DJ (2008). Effect of arginase II on l-arginine depletion and cell growth in murine cell lines of renal cell carcinoma. J Hematol Oncol.

[266] Lemos H, Huang L, Prendergast GC, Mellor AL (2019). Immune control by amino acid catabolism during tumorigenesis and therapy. Nat Rev Cancer.

[267] Sica A, Schioppa T, Mantovani A, Allavena P (2006). Tumour-associated macrophages are a distinct M2 polarised population promoting tumour progression: potential targets of anti-cancer therapy. Eur J Cancer.

[268] Martinez FO, Gordon S, Locati M, Mantovani A (2006). Transcriptional profiling of the human monocyte-to-macrophage differentiation and polarization: new molecules and patterns of gene expression. J Immunol.

[269] Balkwill F, Charles KA, Mantovani A (2005). Smoldering and polarized inflammation in the initiation and promotion of malignant disease. Cancer Cell.

[270] Chang CI, Liao JC, Kuo L (2001). Macrophage arginase promotes tumor cell growth and suppresses nitric oxide-mediated tumor cytotoxicity. Cancer Res.

[271] Davel LE (2002). Arginine metabolic pathways involved in the modulation of tumor-induced angiogenesis by macrophages. FEBS Lett.

[272] Gabrilovich DI (2007). The terminology issue for myeloid-derived suppressor cells. Cancer Res.

[273] Gielen PR (2016). Elevated levels of polymorphonuclear myeloid-derived suppressor cells in patients with glioblastoma highly express S100A8/9 and arginase and suppress T cell function. Neuro Oncol.

[274] Montero AJ, Diaz-Montero CM, Kyriakopoulos CE, Bronte V, Mandruzzato S (2012). Myeloid-derived suppressor cells in cancer patients: a clinical perspective. J Immuno.

[275] Smith C (2012). IDO is a nodal pathogenic driver of lung cancer and metastasis development. Cancer Discov.

[276] Sinha P, Clements VK, Ostrand-Rosenberg S (2005). Interleukin-13-regulated M2 macrophages in combination with myeloid suppressor cells block immune surveillance against metastasis. Cancer Res.

[277] Fultang L, Vardon A, De Santo C, Mussai F (2016). Molecular basis and current strategies of therapeutic arginine depletion for cancer. Internat J Cancer.

[278] Feun L, Savaraj N (2006). Pegylated arginine deiminase: a novel anticancer enzyme agent. Expert Opin Investig Drugs.

[279] Lowery MA (2017). A phase 1/1B trial of ADI-PEG 20 plus nab-paclitaxel and gemcitabine in patients with advanced pancreatic adenocarcinoma. Cancer.

[280] Hernandez CP (2010). Pegylated arginase I: a potential therapeutic approach in T-ALL. Blood.

[281] Steggerda SM (2017). Inhibition of arginase by CB-1158 blocks myeloid cell-mediated immune suppression in the tumor microenvironment. J Immunother Cancer.

